# Enantiomer-dependent biodistribution and exposure determine the *in vivo* behavior of a pleurocidin-derived antimicrobial peptide

**DOI:** 10.1128/mbio.01718-26

**Published:** 2026-08-28

**Authors:** Janis Romanopulos, Paul Cressey, Charlotte K. Hind, Seong-Heun Kim, Zayed A. Shalim, Nadiya Mohamed Akram, Hala Alnasif Alhadi, Amelia Claxton, Yuang You, Anna V. Kelis, Bailey Ramsden, Siham Memdouh, Ana C. Jacob, Tam T. Bui, Simon J. Cleary, Maya Thanou, Richard T. Amison, Vincenzo Abbate, J. Mark Sutton, A. James Mason

**Affiliations:** 1 Institute of Pharmaceutical Science, School of Cancer & Pharmaceutical Sciences, King’s College London, London, United Kingdom; 2 Antimicrobial Discovery, Development and Diagnostics (AD3), Countermeasures Development, Evaluation and Preparedness, UK Health Security Agency 371011, London, United Kingdom; 3 Centre for Biomolecular Spectroscopy and Randall Division of Cell and Molecular Biophysics, King's College London Randall Centre for Cell & Molecular Biophysics 105716, London, United Kingdom; University of California, Davis, Davis, California, USA

**Keywords:** antimicrobial peptides, pharmacokinetics-pharmacodynamics, stereochemistry, biodistribution, immune modulation

## Abstract

Antimicrobial peptides (AMPs) display rapid bactericidal activity *in vitro*, but have largely failed as systemically administered therapeutics, indicating that intrinsic potency alone does not predict *in vivo* efficacy. Here, we examine how pharmacokinetics, stereochemistry, and host context shape early *in vivo* activity using pleurocidin-derived peptides. The D-amino-acid analog D-P-KR rapidly eradicates priority bacterial pathogens within minutes *in vitro*, achieving this at low multiples of the minimum inhibitory concentration (MIC) for *Staphylococcus aureus* but requiring higher multiples for Gram-negative species. Near-infrared imaging of fluorescently labeled enantiomers reveals that stereochemistry strongly influences systemic behavior, with D-enantiomers showing reduced renal and hepatobiliary clearance and increased exposure following intravenous administration. Despite these favorable properties, systemic D-P-KR does not reduce pulmonary bacterial burden within 2 h of treatment in mouse models of *S. aureus* or *Klebsiella pneumoniae* pneumonia. In contrast, increasing exposure through short intravenous infusion produces a rapid, exposure-dependent reduction in pulmonary neutrophil recruitment in *S. aureus* infection, without corresponding early changes in bacterial burden. This effect is not observed in the disseminating *K. pneumoniae* model. These findings demonstrate a dissociation between rapid *in vitro* killing, early bacterial clearance, and host-response modulation, and show that early *in vivo* AMP activity is governed primarily by systemic exposure and infection context. More broadly, this work highlights the need to integrate pharmacokinetics, dosing strategy, and host response when evaluating antimicrobial peptides for systemic use.

**IMPORTANCE:**

Antimicrobial peptides can kill bacteria far more rapidly than most conventional antibiotics, yet very few have succeeded as systemically administered drugs. This study shows that differences in peptide stereochemistry profoundly influence how antimicrobial peptides distribute within the body and how long effective exposure can be maintained. Our findings demonstrate that strong antibacterial activity *in vitro* does not necessarily translate into early bacterial clearance *in vivo*, but that delivery strategies can still affect host responses during infection. By linking peptide stereochemistry, systemic exposure, and biological outcomes, this work highlights why many antimicrobial peptides struggle to translate clinically and underscores the need to prioritize pharmacokinetics, formulation, and dosing strategy alongside antibacterial potency during antimicrobial peptide development.

## INTRODUCTION

Antimicrobial resistance continues to undermine the effectiveness of conventional antibiotics and has intensified efforts to identify alternative therapeutic strategies (1, 2). Antimicrobial peptides (AMPs), which are central components of innate immunity across diverse organisms, have long been attractive candidates because of their broad-spectrum activity, rapid bactericidal effects, and low frequency of resistance emergence (3, 4). *In vitro*, many AMPs kill bacteria within minutes through cooperative interactions with bacterial membranes and intracellular targets (5). Despite this promise, very few AMPs have progressed as systemically administered therapeutics, revealing a persistent disconnect between *in vitro* potency and *in vivo* efficacy (5, 6).

A major contributor to this translational gap is the historical emphasis on antibacterial potency, usually defined by minimum inhibitory concentrations (MIC), at the expense of pharmacokinetic behavior and host–pathogen context (3, 6). AMPs are typically cationic, amphipathic, and conformationally flexible, properties that can promote rapid clearance, non-specific tissue binding, and susceptibility to proteolytic degradation *in vivo* (3, 4). Consequently, concentrations achieved at sites of infection may be transient or insufficient to reproduce the rapid killing observed *in vitro* (6, 7). Moreover, standard susceptibility assays provide little information on the kinetic features of AMP activity, which may be particularly important for understanding both bacterial clearance and host responses (8).

Pleurocidin, a host-defense peptide originally isolated from Winter Flounder, and its synthetic analogs exemplify both the therapeutic potential and challenges of AMPs (9, 10). Pleurocidin-derived peptides display potent activity against a wide range of Gram-positive and Gram-negative pathogens (10) and are capable of killing bacteria on a minute timescale *in vitro*. Our previous work demonstrated that structural modifications, increasing conformational flexibility and peptide–lipid hydrogen bonding, substantially enhance antibacterial potency, most notably against *Staphylococcus aureus* (10). In a mouse lung infection model, we showed that repeated intravenous bolus dosing of the all-d enantiomer pleurocidin analog d-P-KR over 48 h reduced lung bacterial burden, mitigated infection-associated weight loss, and decreased neutrophil recruitment to the lung, with efficacy comparable to vancomycin (10). These findings provided one of the few demonstrations of *in vivo* therapeutic activity for a bactericidal AMP delivered systemically. However, this study also raised important unresolved questions. Efficacy was observed after multiple bolus doses were administered over an extended time window, and this did not enable differentiation of AMPs on the basis of their rapid bactericidal activity (10). Furthermore, the relationship between peptide exposure, tissue distribution, and early biological effects was not defined. Whether d-P-KR achieves sufficient and sustained exposure at the site of infection following systemic administration, how stereochemistry influences its biodistribution, and whether early host responses might be altered independently of bacterial clearance all remain unclear. Addressing these questions is essential for understanding both the limits of current AMP dosing strategies and the potential need for formulation-based approaches.

Incorporation of d-amino acids is widely used in AMP development to improve proteolytic stability, and hence stereochemistry is often assumed to influence *in vivo* behavior primarily through resistance to degradation (11). However, emerging evidence suggests that l- and d-enantiomers may also differ in membrane interactions, cellular uptake, and clearance pathways (11, 12), but direct side-by-side comparisons of whole-body biodistribution remain uncommon (13, 14). Previous AMP biodistribution studies have typically relied on radiolabeling, *ex vivo* tissue homogenization, or indirect pharmacokinetic measurements, approaches that provide limited spatial or temporal resolution and often require assumptions about peptide integrity (15, 16). As a result, the impact of stereochemistry on real-time tissue distribution has remained poorly characterized.

To address this gap, optical imaging using near-infrared (NIR) fluorophores offers a complementary strategy (17). NIR labeling enables longitudinal, whole-body visualization of peptide distribution with high sensitivity and without the need for radioactive tracers (18). Compared with radiotracer-based approaches, such as PET or SPECT, NIR imaging avoids ionizing radiation, permits higher temporal resolution over short timeframes, and is well suited to comparative studies of structurally related peptides, provided that labeling does not perturb biological activity (17). While PET-based approaches can yield absolute quantitative pharmacokinetic data, their application to AMPs is limited by radiochemistry complexity and short isotope half-lives (19). NIR imaging therefore provides a pragmatic but relatively underused approach for interrogating early biodistribution and clearance of AMPs *in vivo*.

In addition to direct antibacterial effects, AMPs are well recognized as modulators of host immune responses (20). Several peptides influence leukocyte recruitment or cytokine production, and these effects may occur on a different timescale from bacterial killing (20–22). In the context of acute lung infection, early modulation of neutrophil recruitment could influence tissue damage, disease severity, and subsequent bacterial clearance, even in the absence of immediate reductions in bacterial burden (23–26). Disentangling direct antibacterial activity from host-mediated effects therefore requires careful attention to timing, exposure, and infection context.

In the present study, we build directly on our previous findings by investigating how pharmacokinetics-pharmacodynamics, stereochemistry, and delivery strategy shape the early *in vivo* behavior of the pleurocidin-derived peptide d-P-KR (10). We combine detailed *in vitro* time-kill analyses with NIR-based whole-body imaging of l- and d-enantiomers to define stereochemistry-dependent biodistribution and clearance following systemic administration. We then examine how bolus versus infusion dosing influences early outcomes in mouse lung infection models caused by *Staphylococcus aureus* and *Klebsiella pneumoniae*, two pathogens with distinct inflammatory kinetics and dissemination potential (23–26). By focusing on early time points, this work seeks to clarify why potent *in vitro* activity and longer-term efficacy do not necessarily translate into immediate bacterial clearance, and to identify principles that can inform future formulation- and exposure-driven optimization of antimicrobial peptides.

## MATERIALS AND METHODS

### Peptide synthesis and fluorescent labeling

Peptides (pleurocidin, d-pleurocidin, d-P-KR, and labeled analogs) were prepared by automated Fmoc solid-phase peptide synthesis on Rink amide MBHA LL resin using standard N,N′-diisopropylcarbodiimide/Oxyma coupling and piperidine deprotection using a Biotage Initiator+ Alstra synthesizer. According to a scheme based on previous methods (Scheme S1) (27, 28), 6-carboxyfluorescein (6-FAM) was coupled on-resin, and selective 1-(4,4-dimethyl-2,6-dioxacyclohexylidene)ethyl deprotection was performed using hydroxylamine/imidazole conditions. For NIR imaging, IRDye 800CW NHS ester was coupled on-resin to the C-terminus via a lysine linker under anhydrous conditions (Scheme S2). Peptides were cleaved and side-chain deprotected using trifluoroacetic acid-based cocktails, precipitated in cold ether, and purified by preparative reverse-phase HPLC using an Agilent 1100 Series HPLC system and a Waters Symmetry C8 Prep column (inner diameter—19 mm, length—300 mm, particle size—7 μm, spherical, pore size—100 Å). Final purity was assessed by analytical RP-HPLC (214 nm), and identities confirmed by mass spectrometry (MS) using a Waters MicroMass ZQ quadrupole mass analyzer with electrospray ionization in the positive mode; selected peptides were additionally verified by high-resolution MS using a Thermo Scientific Q Exactive Hybrid Quadrupole-Orbitrap operating under electrospray ionization in the positive mode.

### *In silico* calculation of physicochemical properties

The number of hydrogen bond acceptors (HBAs) and hydrogen bond donors (HBDs) were calculated using openmolecules.org DataWarrior (29). The pI, clogP, clogD at pH 7.4, and the average charge at pH 7.4 were calculated using Chemaxon MarvinSketch (Certara, Radnor, PA).

### Bacterial strains and growth conditions

Strains are listed in the text and supplemental tables. Overnight cultures were grown in tryptic soy broth at 37°C with shaking. Where indicated, assays were performed in Mueller-Hinton broth (MHB) or RPMI 1640 supplemented with 5% MHB (RPMI).

### Minimum inhibitory concentrations

MICs were determined by broth microdilution as previously described (8, 10), using twofold serial dilutions of peptide in 96-well polypropylene plates and an initial inoculum equivalent to OD_600_ 0.005. Plates were incubated statically at 37°C overnight, then OD_600_ readings were taken with a BMG Labtech FLUOstar Omega microplate reader, and MIC was defined as the lowest concentration preventing growth (OD_600_ < 0.1).

### *In vitro* pharmacodynamics (time-kill)

Time-kill assays were based on Clarke et al. (30) but with increased early sampling to capture rapid bactericidal activity. Bacteria (5 × 10^6^ CFU/mL) were exposed to serial peptide concentrations (initially up to 32× MIC; extended to 512× MIC when required). Aliquots were removed at baseline and selected time points, serially diluted in phosphate-buffered saline (PBS) and plated on tryptic soy agar (TSA) for colony enumeration. Regrowth was assessed by continued incubation and sampling at 24 and 48 h.

### Mammalian cell culture and MTT cytotoxicity

A549, HepG2, and HEK293T cells were maintained in Dulbecco’s modified Eagle’s medium supplemented with 10% heat-inactivated fetal bovine serum and 1% penicillin/streptomycin at 37°C/5% CO_2_. For MTT assays, cells were seeded (2 × 10^4^ cells/well), exposed to twofold serial peptide dilutions for the specified duration, and metabolic activity quantified by MTT reduction with absorbance readout at 550 nm in a Tecan Infinite 200 PRO microplate reader.

### Circular dichroism spectroscopy

Far-UV CD spectra (50 µM peptide) were recorded in 0.5 mm cuvettes from 185 to 260 nm (bandwidth—2 nm, step—1 nm, time per point—1.2 s) using an Applied Photophysics Chirascan Plus spectrometer. Temperature ramp experiments were performed by heating from 6°C at 1°C/min with spectra collected approximately every 2°C. The sample was then cooled to 23°C and an additional spectrum acquired. Solvent baselines were subtracted, and spectra smoothed using a Savitzky–Golay filter (window size 5 points).

### NIR biodistribution imaging

Female BALB/c mice (6–8 weeks) received either an intravenous bolus dose (2.5 mg/kg; ~100 µL of 0.5 mg/mL peptide in saline) or a short intravenous infusion (10 mg/kg; ~200 µL of 1 mg/mL peptide in saline delivered over 30 min via tail vein cannulation and syringe pump). The day prior to imaging, the mice were put under isoflurane anesthesia and shaved apart from their heads. The shaved areas were treated with hair-removal cream and cleaned. Infusions were performed using an infusion kit consisting of a Mesorelle 30G 1-inch needle, 3 cm piece of BTPE-10 polyethylene tubing, 3 cm piece of BTPU-040 polyurethane tubing, and PNP3F22 PinPort connected to each other in this order, with each joint being secured by superglue. Infusion solutions were withdrawn into 1 mL syringes, which were then docked into a Harvard Apparatus Pump 11 Elite syringe pump. Each infusion kit was sterilized with 70% ethanol in water and pre-filled with sterile saline before cannulation. Once successful cannulation was performed, the infusion kits were secured in the injection site by surgical glue and connected to the infusion-solution-containing syringes via a PNP3MC/25-18 PinPort extension set (Instech Laboratories) pre-filled with the same infusion solution. The administration of the infusion solution was started immediately after the connection had been made by operating the syringe pump at the appropriate flow rate for the intended duration.

Whole-body fluorescence imaging was performed under isoflurane anesthesia using a CRi Maestro EX system (excitation 684–729 nm; emission 740–950 nm; acquired in 10 nm increments; exposure time—100 ms). Animals were imaged pre-dose and at defined time points; organs were excised and imaged at the terminal time point. Spectral unmixing was performed in Maestro software using a reference spectrum obtained from labeled peptide in saline. Region-of-interest intensities were quantified in ImageJ with background subtraction. Where dorsal images were available, signal from the lumbar region was used as a blood proxy for normalization; when ventral-only imaging was performed, signal from the thoracic region was used for normalization.

### Mouse lung infection models

Male C57BL/6J mice (8–10 weeks) were infected oropharyngeally (50 µL) under isoflurane. The 3 h EMRSA-15 agar-bead model followed Manzo et al. (10) using agar bead preparation based on established chronic airway infection methods (31). After 1 h, the animals were injected with saline (vehicle control) or a 0.75 mg/mL solution of d-P-KR in sterile saline (2.5 mg/kg, ~100 µL) into the tail vein or anaesthetized with isoflurane and—as for the biodistribution study—infused with a 1.5 mg/mL solution of d-P-KR in sterile saline over 30 min (10 mg/kg, ~200 µL). For the 26 h delafloxacin-resistant *K. pneumoniae* M6 model, mice received diluted bacterial suspension oropharyngeally (equivalent to 1 × 10^6^ CFU of delafloxacin-resistant *K. pneumoniae* M6) under isoflurane anesthesia and were treated 24 h later with vehicle, gentamicin (50 mg/kg i.v.), or d-P-KR (2.5 mg/kg bolus or 10 mg/kg 30 min infusion) and culled 2 h after treatment initiation.

### Sample collection and BAL differential counts

BAL was performed with 3 × 0.5 mL PBS via tracheal cannulation. Lungs and selected organs were aseptically excised, homogenized in PBS, serially diluted, and plated on TSA for CFU enumeration. Total BAL leukocytes were counted using Turk’s solution and a hemocytometer. Differential counts were obtained from cytospin preparations stained with Kwik-Diff, counting 200 cells per slide to determine neutrophil and monocyte percentages.

### Cytokine analysis

BAL samples were diluted 1:2 in PBS (Oxoid, BR0014G) before analysis. Plasma samples were diluted in PBS according to analyte: CRP, 1:1,000; IL-10 and MCP-1, 1:100; and TNF-α, 1:10. Cytokine concentrations were measured using R&D Systems DuoSet ELISA Development Kits according to the manufacturer’s instructions: CRP (DY1829), TNF-α (DY410), IL-1β (DY401), IL-6 (DY406), IL-10 (DY417), and MCP-1 (DY479). Assays were performed using DuoSet ELISA Ancillary Reagent Kit 2 (R&D Systems, DY008B), except for IL-10 and MCP-1 assays in BAL and plasma, which were performed in Greiner CELLSTAR 96-well cell culture plates (655180) rather than standard ELISA plates. Sandwich ELISAs were performed according to the general DuoSet protocol. Plates were coated overnight at room temperature with capture antibody diluted in PBS without carrier protein, washed three times, and blocked with reagent diluent (1% BSA in PBS) for 1 h at room temperature. Standards were prepared as twofold serial dilutions and assayed in duplicate. Diluted samples and standards (100 µL/well) were added and incubated for 2 h at room temperature, followed by detection antibody for 2 h. After washing, plates were incubated with streptavidin-HRP for 20 min in the dark, developed with TMB substrate for 20 min in the dark, and the reaction was stopped with 50 µL/well sulfuric acid stop solution. Absorbance was measured immediately at 450 nm with wavelength correction at 540 nm using a SpectraMax Plus 384 microplate reader (Molecular Devices). Standard curves were fitted using 4-parameter logistic regression, and sample concentrations were interpolated, corrected for dilution, and background-subtracted using the zero standard.

### Statistical analysis

Cytokine concentrations and log_10_-transformed CFU and BAL leukocyte counts are presented as mean ± SEM. Group comparisons used one-way ANOVA with Dunnett’s correction or Kruskal–Wallis with Dunn’s correction, depending on normality and variance assumptions (Shapiro–Wilk test).

## RESULTS

The pleurocidin-derived peptide d-P-KR has potent activity against *Staphylococcus aureus* and *Klebsiella pneumoniae*, with minimal inhibitory concentrations in the low micromolar range (10). Here (Tables S1 through S3), we extend the antimicrobial testing to include a larger number of *Pseudomonas aeruginosa* isolates, as well as evaluating performance in RPMI 1640 media supplemented with 5% MHB (RPMI), which has been suggested to have increased physiological relevance compared with MHB (32). MICs obtained for pleurocidin, d-pleurocidin, and d-P-KR are consistent with those obtained previously (10). MICs for pleurocidin and d-pleurocidin are similar in MHB with the exception of those for *K. pneumoniae* and *P. aeruginosa*, which are consistently lower for the d-enantiomer (Table S1).

In contrast, d-pleurocidin substantially outperforms pleurocidin when RPMI media is used; indeed, almost all *P. aeruginosa* isolates are insensitive to pleurocidin in the concentration range tested, while MICs for *S. aureus* and *K. pneumoniae* isolates are approximately fourfold lower for the d-enantiomer (Table S2). Under these more exacting conditions, d-P-KR outperforms d-pleurocidin (Table S3).

**Fig 1:**
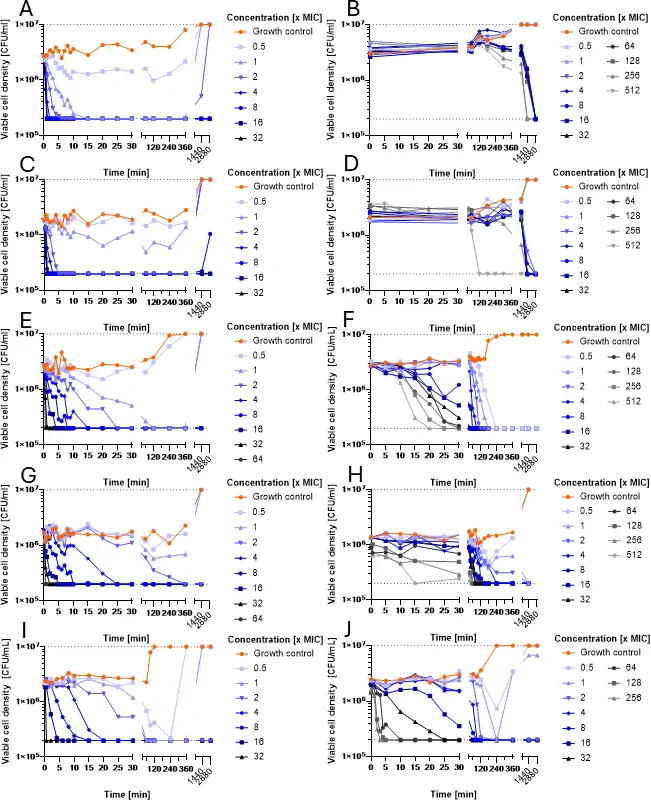
d-P-KR is rapidly bactericidal *in vitro*. Time-kill curves, representative of three independent replicates, are shown for the following antibiotic–bacteria–media combinations: d-P-KR (**A**) and tigecycline (**B**) against *S. aureus* NCTC 13616 (EMRSA-15) in MHB; d-P-KR (**C**) and tigecycline (**D**) against *S. aureus* NCTC 13616 (EMRSA-15) in RPMI (5% MHB); d-P-KR (**E**) and gentamicin (**F**) against *A. baumannii* ATCC 17978 in RPMI (5% MHB); d-P-KR (**G**) and tobramycin (**H**) against *P. aeruginosa* RP73 in RPMI (5% MHB); d-P-KR (**I**) and gentamicin (**J**) against *K. pneumoniae* M6 in RPMI (5% MHB). Curves are for indicated multiples of the MIC (Table 1). Error bars not shown for clarity. Limit of detection is 2 × 10^5^ CFU/mL in each case.

To establish the intrinsic pharmacodynamic properties of d-P-KR, we assessed its time-dependent antibacterial activity against selected, clinically relevant pathogens (Fig. 1) and compared this with tigecycline (Fig. 1B and D), considered bacteriostatic (33), and the aminoglycosides tobramycin (Fig. 1H) and gentamicin (Fig. 1F and J), considered among the most rapidly bactericidal clinically used antibiotics (Table 1) (34). Consistent with a membrane-active mechanism, time-kill assays reveal rapid, concentration-dependent bactericidal activity. At low multiples of the MIC, d-P-KR reduces viable *S. aureus* counts within minutes irrespective of the media used (Fig. 1A and C). In contrast, higher multiples of the MIC are required to achieve comparable rates of killing against *K. pneumoniae*, likely reflecting the additional permeability barriers associated with the Gram-negative outer membrane (Fig. 1I). Nevertheless, near-complete killing is still achieved on a timescale substantially faster than that typically observed for conventional bactericidal antibiotics, such as gentamicin, and with a lower multiple of the MIC (Fig. 1J). To determine whether this behavior extended to other Gram-negative pathogens, time-kill assays were additionally performed against *Acinetobacter baumannii* and *Pseudomonas aeruginosa*. For both species, d-P-KR again exhibits rapid bactericidal activity once a threshold concentration is exceeded, with marked reductions in viable counts occurring within the first few minutes of exposure (Fig. 1E and G). However, as observed for *K. pneumoniae*, these rapid killing kinetics required higher peptide concentrations relative to those effective against *S. aureus* (Table 1).

**TABLE 1:** *In vitro* pharmacodynamic comparison of d-P-KR and classical antibiotics^*a*^

Condition		Lowest multiple of MIC to reduce bacterial load below limit of detection			
Isolate (medium)	Antimicrobial (MIC, µg/mL)	5 min	30 min	6 h	24 h
*S. aureus* NCTC 13616 (MHB)	d-P-KR (8)	4	1	1	4^*b*^
	Tigecycline (0.5)	–^*c*^	–	–	8
*S. aureus* NCTC 13616 (RPMI)	d-P-KR (2)	2	2	2	16^*b*^
	Tigecycline (2)	–	–	512	8
*A. baumannii* ATCC 17978 (RPMI)	d-P-KR (2)	16	2	1	4^*b*^
	Tigecycline (0.25)	–	–	4	1
	Gentamicin (1)	–	128	0.5	0.5
*P. aeruginosa* RP73 (RPMI)	d-P-KR (16)	16	4	2	2
	Tigecycline (8)	256	128	32	1
	Tobramycin (16)	–	–	4	2
*K. pneumoniae* M6 (delafloxacin resistant) (RPMI)	d-P-KR (4)	16	4	0.5	0.5
	Gentamicin (0.25)	128	32	0.5	0.5

^*a*^ The minimal MIC multiples of d-P-KR, tigecycline, or aminoglycoside antibiotics necessary to reduce the bacterial load of various isolates below the limit of detection within selected times in either RPMI (5% MHB) or MHB are given.

^*b*^ Conditions affected by regrowth.

^*c*^ “–” indicates that no tested concentration led to a bacterial load reduction below the limit of detection.

Consistent with the known greater potency of aminoglycosides against *Enterobacteriaceae* (34), *K. pneumoniae* is both more sensitive and succumbs more quickly (at comparable MIC multiples) to gentamicin than either *A. baumannii* or *P. aeruginosa* are to gentamicin and tobramycin.

Together, these data demonstrate that d-P-KR is capable of rapid bactericidal activity across multiple bacterial species, but that the concentration required to achieve fast killing is strongly influenced by bacterial envelope architecture. In particular, Gram-negative pathogens consistently require higher peptide exposures to achieve minute-scale killing, although d-P-KR still outperforms aminoglycosides in this respect. These findings establish a robust pharmacodynamic framework for interpreting subsequent *in vivo* studies, in which systemic exposure rather than intrinsic antibacterial activity may be rate-limiting. Analogously, we also considered the time-resolved toxicity of d-P-KR toward three human cell lines *in vitro* (Table 2 and Fig. S1) in case there are temporal differences in toxicity that might be exploited or which might compound longer exposure durations *in vivo*. We find no differences in toxicity toward A549 (lung), HEK293T (kidney), or HepG2 (liver) cells, representing the target and clearance organs, respectively, between exposures of 30 min, 1 h, 4 h, or 24 h. Notably, however, the approximate 10-fold selectivity window (based on IC_50_ to MIC ratios) is eroded if the higher concentrations needed for fast killing are considered.

**TABLE 2:** Time-resolved cytotoxicity of d-P-KR^*a*^

Cell line	IC_50_ ± SEM (µg/mL)			
	0.5 h	1 h	4 h	24 h
A549	48.7 ± 1.7	47.9 ± 0.8	42.5 ± 1.1	45.6 ± 0.9
HEK293T	30.3 ± 3.3	28.1 ± 2.2	24.7 ± 2.6	29.8 ±^*b*^
Hep G2	47.5 ± 3.4	46.5 ± 2.2	39.7 ± 3.8	41.4 ± 1.9

^*a*^ The IC_50_ values for A549 (lung), HEK 293T (kidney), and HepG2 (liver) cells treated with d-P-KR for 0.5, 1, 4, and 24 h. The values are presented as the best-fit values for the curves presented in Fig. S1 ± SEM.

^*b*^ Unstable fit prevents error estimation.

We next examined whether peptide stereochemistry influenced systemic distribution and clearance *in vivo*. Because fluorescent labeling has the potential to alter peptide physicochemical properties, conformation, and biological activity, we first characterized the labeled analogs prior to *in vivo* imaging. Table 3 summarizes the sequences and calculated physicochemical properties of the parent peptides and their fluorescent analogs. Incorporation of either 6-FAM at either the N- or C-terminus or IRDye 800CW at the C-terminus increases molecular size, hydrogen-bonding capacity, and lipophilicity while largely preserving the predicted net positive charge at physiological pH. We then evaluated the experimental consequences of fluorescent labeling by determining antibacterial activity and secondary structure. Peptides were labeled with a 6-FAM fluorescent tag, and antibacterial activity was evaluated by MIC determination alongside conformational analysis by circular dichroism (CD) spectroscopy. Across all three peptide backbones, C-terminal labeling was better tolerated with smaller MIC shifts and minimal perturbation of peptide conformation (Tables S1 through S3 and Fig. S2 through S6). CD spectra revealed that C-terminally 6-FAM-labeled peptides retained the characteristic α-helix content observed for their unlabeled counterparts in the presence of trifluoroethanol or SDS micelles, as a simple membrane-mimicking medium, while a mixture of disordered and P_II_ conformation is observed in 5 mM Tris buffer, which is identical to the unlabeled peptide (Fig. S2 through S5). For d-P-KR, some loss of P_II_ conformations is observed in 5 mM Tris buffer upon labeling with 6-FAM (Fig. S3 and S6), but the conformation in SDS is unaffected.

**TABLE 3:** Sequences and calculated physicochemical properties of pleurocidin-derived peptides and their fluorescent analogs^*a*^

Peptide	Sequence	HBA	HBD	Charge (pH 7.4)	pI	clogP	clogD (pH 7.4)
Pleurocidin (P)	GWGSFFKKAAHVGKHVGKAALTHYL-NH_2_	65	37	5.93	10.84	−10.35	−21.58
KFP	**K**(**6-FAM**)GWGSFFKKAAHVGKHVGKAALTHYL-NH_2_	74	41	4.33	10.11	−8.31	−18.99
PKF	GWGSFFKKAAHVGKHVGKAALTHYL**K**(**6-FAM**)-NH_2_	74	41	4.17	10.11	−8.31	−18.76
P-KR	GWGSFFRRAAHVGRHVGRAALTHYL-NH_2_	73	45	5.90	11.73	−13.46	−23.93
PKF-KR	GWGSFFRRAAHVGRHVGRAALTHYL**K**(**6-FAM**)-NH_2_	82	49	3.93	11.33	−11.42	−21.04
PKI-KR	GWGSFFRRAAHVGRHVGRAALTHYL**K**(**IRDye**)-NH_2_	91	51	2.63	10.35	−10.37	−11.94

^*a*^ All peptides were amidated at the C-terminus, giving an overall increase in nominal charge of +1. All d-amino acid enantiomers share the same sequences and biophysical characteristics as their corresponding l-enantiomers. Fluorescent tag highlighted in bold in the peptide sequences.

**Fig 2:**
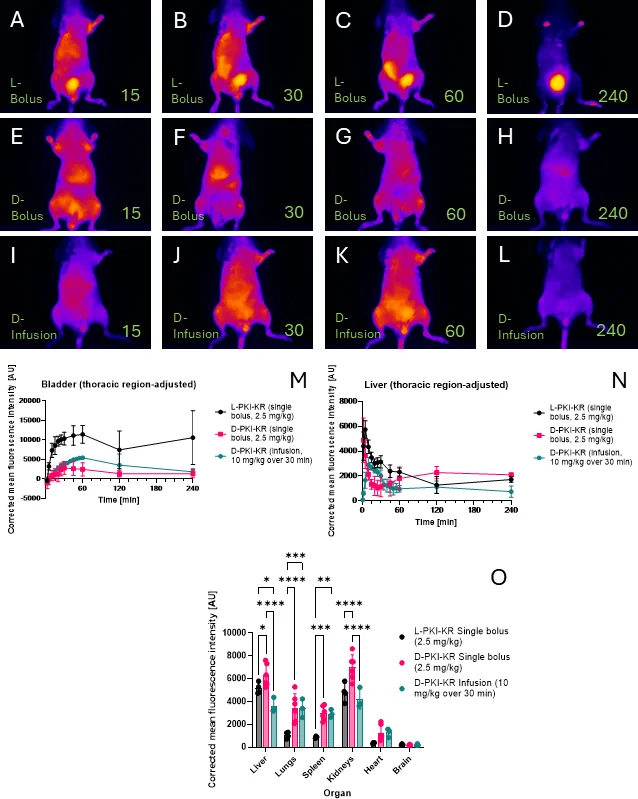
Peptide stereochemistry and delivery rate determine systemic biodistribution following intravenous administration**.** Representative ventral images of the biodistribution at selected time points of IRDye 800CW labeled pleurocidin-KR (**A–D**) or d-P-KR (**E–L**) in female BALB-c mice administered as either a 2.5 mg/kg bolus (**A–H**) or a 10 mg/kg infusion (**I–L**) over 30 min. Corrected mean ± SD fluorescence intensity for areas corresponding to bladder (**M**) and liver (**N**), and dissected organs at *t* = 240 min (**O**). Two-way ANOVA with Tukey multiple comparisons test. *P ≤ 0.05; **P ≤ 0.01; ***P ≤ 0.001; ****P ≤ 0.0001.

**Fig 3:**
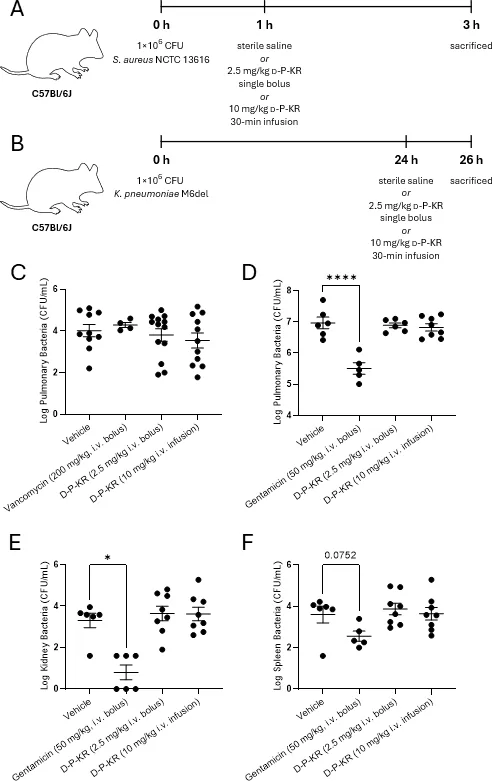
Early antibacterial efficacy in mouse pneumonia models**.** Study designs (**A and B**) and therapy of C57Bl/6 mice at 3 h p.i. infected with *S. aureus* NCTC 13616 (EMRSA-15) (**C**) or at 26 h p.i. infected with delafloxacin-resistant *K. pneumoniae* (**D–F**), as revealed by CFU/mL in each tissue. Comparisons are Lognormal ordinary one-way ANOVA (**D**) or Kruskal-Wallis (**E and F**), according to data normality and/or assumption of equal SD, with only comparisons *P* ≤ 0.1 shown. ****P ≤ 0.0001, *P ≤ 0.05. Data are shown as mean ± SEM.

**Fig 4:**
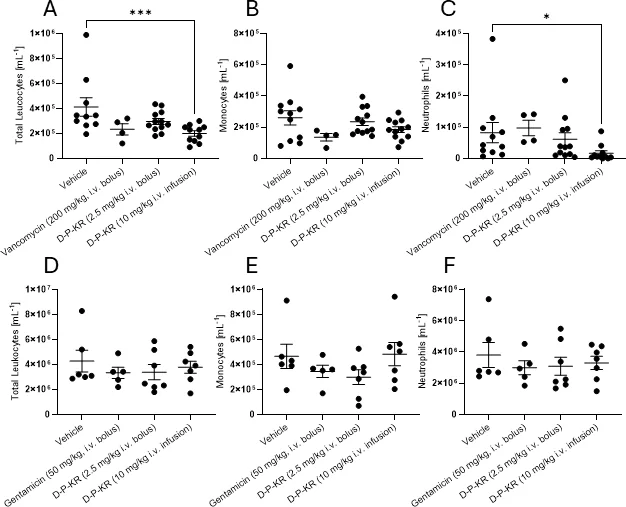
Exposure-dependent reduction in pulmonary leukocyte recruitment in *Staphylococcus aureus* pneumonia with divergent inflammatory responses in *Klebsiella pneumoniae* infection. BAL immune cell response to therapy of C57Bl/6 mice at 3 h p.i. infected with EMRSA-15 (**A–-C**) or at 26 h p.i. infected with delafloxacin-resistant *K. pneumoniae* (**D–F**). Comparisons are Lognormal ordinary one-way ANOVA (**A**), Brown-Forsythe and Welch ANOVA, or Kruskal-Wallis (**C**) test according to data normality and/or assumption of equal SD, with only comparisons *P* ≤ 0.05 shown. ***P ≤ 0.001; *P ≤ 0.05. Data are shown as mean ± SEM.

These experiments were intended to identify the labeling position least likely to perturb peptide behavior rather than to demonstrate that fluorescently labeled conjugates fully reproduce the properties of the parent peptide. This optimization strategy was then carried forward for near-infrared imaging studies, in which C-terminal IRDye 800CW conjugates were used as matched imaging probes to compare the relative *in vivo* behavior of the l- and d-enantiomers. The 6-FAM data serve as a practical proxy for tolerance of the labeling position when selecting the corresponding C-terminal IRDye 800CW conjugates for biodistribution studies. Both fluorophores increase molecular size, hydrogen-bonding capacity, and lipophilicity while largely preserving the overall cationic character of the parent peptides, although the magnitude of these changes differs between the two labels (Table 3). Furthermore, while the two IRDye 800CW conjugates are necessarily distinct molecular entities, use of identical conjugation chemistry allowed stereochemistry to be examined under directly comparable conditions. IRDye 800CW was selected to enable sensitive, longitudinal whole-body imaging while avoiding tissue autofluorescence and the need for radioactive tracers. Although the physicochemical properties of the labeled analogs necessarily differ from those of the parent peptides (Table 3), the use of chemically matched C-terminal IRDye conjugates allowed direct comparison of stereochemistry-dependent biodistribution under identical labeling conditions. Having established that C-terminal near-infrared labeling does not materially alter peptide conformation in SDS micelles (Fig. S4 and S7), we next used this strategy to investigate stereochemistry-dependent biodistribution and clearance *in vivo*.

Whole-body imaging reveals clear and reproducible differences in biodistribution between the two enantiomers when administered as a single intravenous bolus dose of 2.5 mg/kg (Fig. 2). Following administration, the IRDye-labeled l-enantiomer and/or its fluorescent metabolites are rapidly cleared from the circulation, with signal accumulating initially in the kidneys and liver and soon after in the bladder and small intestine, indicating extensive renal and hepatobiliary clearance (Fig. 2A through D, M, and N). In contrast, the IRDye-labeled d-enantiomer analog and/or its fluorescent metabolites display a more sustained systemic signal, with faster redistribution from the liver and prolonged whole-body retention (Fig. 2E through H, M, and N). There is also no evidence for hepatobiliary clearance in the case of the d-enantiomer. Quantitative analysis of excised organ signal intensities confirms significantly slower clearance kinetics for the d-enantiomer compared with its l-counterpart across multiple organs, including the lung (Fig. 2O). These data demonstrate that stereochemistry alone is sufficient to markedly alter the *in vivo* disposition of pleurocidin-derived peptides following systemic administration. Importantly, these differences extend beyond proteolytic stability and indicate that the enantiomeric form influences whole-body exposure, clearance mechanism, and tissue distribution on early time scales relevant to infection intervention.

Given the rapid clearance observed following intravenous bolus administration, we next examined whether systemic exposure could be increased by modifying both dose and delivery strategy. To address this, the d-enantiomer was additionally administered as a short, 30 min intravenous infusion delivering a higher total dose (10 mg/kg). Whole-body near-infrared imaging was then used to compare peptide distribution and clearance kinetics over early time points. While bolus administration at 2.5 mg/kg produces a transient systemic signal that declines rapidly, infusion of 10 mg/kg results in a substantially higher and more sustained whole-body signal, with prolonged retention in both central and peripheral compartments (Fig. 2I through L).

Notably, despite the higher total dose delivered by infusion, early signal intensities in clearance organs, such as the liver and kidney, are not increased (Fig. 2M and N), and at 4 h post-administration onset, are significantly lower than those observed following bolus administration (*P* < 0.0001), even while the residual intensities in the lungs are equivalent (Fig. 2O). This pattern is consistent with differences in peak exposure and delivery kinetics between bolus and infusion dosing, rather than reduced overall tissue exposure. Together, these data indicate that both dose and delivery strategy critically influence early systemic exposure of pleurocidin-derived peptides and determine their initial biodistribution following intravenous administration.

Having established that d-P-KR exhibits rapid bactericidal activity *in vitro* and that systemic exposure is strongly influenced by stereochemistry, dose, and delivery strategy, we next assessed early antibacterial efficacy *in vivo* using mouse lung infection models caused by *Staphylococcus aureus* and *Klebsiella pneumoniae*.

In the early-intervention *S. aureus*/EMRSA-15 pneumonia model, treatment was initiated 1 h after infection, and lung bacterial burden was quantified 2 h after the onset of dosing. In the absence of treatment, a somewhat variable lung burden of 4.0 ± 0.3 log_10_ CFU/mL is obtained (Fig. 3C). Within this early time window, vancomycin does not produce a measurable reduction in pulmonary colony-forming units relative to vehicle-treated controls (Fig. 3C). This is consistent with the primarily bacteriostatic activity of vancomycin against the EMRSA-15 strain used in this model and indicates that detectable reductions in bacterial burden are not expected at this stage of infection. Under the same conditions, d-P-KR administered as either a lower-dose intravenous bolus (2.5 mg/kg) or a higher-dose short intravenous infusion (10 mg/kg) likewise does not produce a statistically significant reduction in lung bacterial burden (Fig. 3C).

We next evaluated antibacterial efficacy in a disseminating *K. pneumoniae* lung infection model, in which a higher lung burden (7.0 ± 0.2 log_10_ CFU/mL) is established after 24 h of infection, and bacteria spread to extrapulmonary organs, including the spleen and kidney (Fig. 3D through F). In contrast to vancomycin in the *S. aureus* model, gentamicin treatment, at a dose close to the maximum tolerated level, produces a marked reduction in bacterial burden across pulmonary and systemic compartments within the same early post-treatment interval (Fig. 3D through F). This finding is consistent with the rapid bactericidal activity of aminoglycosides observed *in vitro* and confirms that the 2 h time point is sufficient to detect early antibacterial effects when bactericidal exposure is achieved. Under identical conditions, however, d-P-KR administration does not significantly reduce bacterial counts in the lung, spleen, or kidney, regardless of dosing strategy.

Together, these findings indicate that systemic administration of d-P-KR does not produce detectable reductions in bacterial burden within the first 2 h of treatment in either localized *S. aureus* pneumonia or disseminating *K. pneumoniae* infection. When interpreted alongside the rapid *in vitro* bactericidal kinetics and exposure-dependent biodistribution, these data are consistent with early *in vivo* antibacterial efficacy of d-P-KR being constrained primarily by pharmacokinetic and tissue-exposure factors rather than intrinsic antimicrobial potency, and that measurable reductions in bacterial burden may not be expected until sufficient exposure is achieved.

Because reductions in bacterial burden are not detected within the early post-treatment interval, we next asked whether systemic administration of d-P-KR influenced host inflammatory responses during the same time window. Bronchoalveolar lavage fluid (BALF) was collected from mice in the early-intervention EMRSA-15 pneumonia model, and total leukocytes, together with monocyte and neutrophil populations, were quantified 2 h after the onset of treatment. Administration of d-P-KR as a short intravenous infusion (10 mg/kg) produces a significant reduction in total BALF leukocyte numbers compared with vehicle-treated controls (*P* < 0.001), whereas the lower-dose bolus (2.5 mg/kg) has no significant effect (Fig. 4A). Monocyte counts are not significantly altered by d-P-KR under either dosing regimen (Fig. 4B). In contrast, neutrophil recruitment to the airways is significantly reduced following the higher-dose infusion but not after bolus administration (*P* < 0.05) (Fig. 4C).

Importantly, these exposure-dependent reductions in pulmonary leukocyte and neutrophil numbers occur in the absence of measurable decreases in pulmonary bacterial burden at the same time point (Fig. 3C), indicating that early changes in inflammatory cell recruitment can be temporally dissociated from detectable antibacterial effects. While this is most simply interpreted as altered recruitment to the airways, alternative explanations—including changes in neutrophil activation state, intravascular retention, or peptide-induced processes, such as neutrophil extracellular trap formation—cannot be excluded at this stage.

We next assessed inflammatory cell recruitment in the disseminating *Klebsiella pneumoniae* infection model. In contrast to the clear exposure-dependent reductions observed in EMRSA-15 pneumonia, d-P-KR administration does not produce comparable decreases in BALF leukocyte or neutrophil numbers within the same early post-treatment interval (Fig. 4D through F). These findings indicate that early host-response effects of systemic d-P-KR differ between infection contexts and suggest that pathogen-specific inflammatory dynamics may influence the pulmonary cellular response to peptide exposure.

Consistent with the modest and context-dependent changes observed in inflammatory cell recruitment, cytokine responses measured in BAL fluid and plasma showed limited and variable modulation across conditions (Fig. S8 through S11), supporting the view that early host-response effects are not dominated by a single cytokine pathway.

## DISCUSSION

In this study, we investigated how pharmacokinetics, stereochemistry, and delivery strategy affect the early *in vivo* behavior of the pleurocidin-derived antimicrobial peptide d-P-KR. Consistent with a membrane-active mechanism, d-P-KR displayed rapid, concentration-dependent bactericidal activity across multiple bacterial species *in vitro*. Whole-body imaging of matched IRDye-labeled analogs revealed that systemic exposure following intravenous administration was strongly influenced by peptide stereochemistry, dose, and delivery kinetics. Despite this rapid intrinsic antibacterial activity, systemic administration did not reduce bacterial burden within the first 2 h of treatment in mouse models of *Staphylococcus aureus* (EMRSA-15) or disseminating *Klebsiella pneumoniae* infection. However, sufficiently high exposure achieved by short intravenous infusion produced rapid, exposure-dependent reductions in pulmonary leukocyte and neutrophil recruitment during acute EMRSA-15 infection, an effect that was not observed in the *Klebsiella* model. Together, these findings demonstrate a temporal dissociation between early host-response modulation and detectable antibacterial efficacy, and indicate that early *in vivo* activity of systemically delivered antimicrobial peptides is governed primarily by exposure kinetics and infection context rather than intrinsic bactericidal potency alone. The implications of these observations for interpreting early efficacy readouts, understanding pathogen-specific inflammatory dynamics, and guiding exposure-optimized or formulation-based development strategies are considered below.

A central question raised by our data is why early reductions in pulmonary CFU were not detected in the EMRSA-15 agar-bead model at 2 h after treatment onset (3 h p.i.), despite the rapid *in vitro* killing kinetics of d-P-KR and prior evidence of efficacy with repeated bolus dosing over 48 h (10). One straightforward explanation is that this early sampling point is not well matched to the biology of bacterial clearance *in vivo*. Even for rapid bactericidal agents, reductions in recoverable CFU can lag behind exposure because bacteria may be sublethally damaged or transiently growth-arrested, killing may occur within microenvironments not well captured by bulk homogenate CFU, and physical clearance of killed bacteria takes time. Here, the 3 h p.i. lung burden was substantially lower than at 48 h p.i. (10), consistent with the notion that this is a very early phase of infection in which CFU dynamics may be relatively insensitive to short exposure windows. The agar-bead format may further decouple early systemic exposure from measurable CFU change by creating diffusion barriers and biofilm-like protection, increasing the importance of time above an effective concentration and/or repeated exposures—an issue well recognized in chronic airway infection modeling (35). Importantly, dissociation between early CFU and inflammatory readouts is also plausible. Early pneumonia is characterized by rapid cytokine induction (e.g., TNF-α, KC/CXCL1, MIP-2/CXCL2) within ~30 min p.i., followed by neutrophil influx over the ensuing hours, and changes in inflammatory mediators (or neutrophil activity markers) may be detectable earlier than changes in BAL cell counts (25, 36, 37). Together, these considerations suggest that lack of early CFU reduction does not contradict longer-term efficacy. Instead, it highlights the need to align CFU and immune readouts with exposure kinetics and with the physical/inflammatory structure of the infection niche (25, 36, 37).

The contrasting antibacterial and inflammatory responses observed in the EMRSA-15 and *Klebsiella pneumoniae* models underline the importance of infection context in determining early *in vivo* activity of systemically delivered antimicrobial peptides. Although d-P-KR displayed rapid, concentration-dependent killing across species *in vitro*, substantially higher multiples of the MIC were required to achieve minute-scale bactericidal activity against Gram-negative organisms in these assays, consistent with the added permeability/interaction constraints of the Gram-negative envelope and the broader mechanistic framework for AMP action at membranes (38–40). This more exacting concentration requirement provides a plausible explanation for the absence of early antibacterial effects in the disseminating *Klebsiella* model despite the clear efficacy of gentamicin, a rapidly bactericidal antibiotic for which killing is strongly concentration-dependent at high exposure (41). Differences intrinsic to the infection models are also likely to contribute since *K. pneumoniae* disease biology varies substantially by lineage and virulence determinants and can include high bacterial burden, intense neutrophil-driven inflammation, and dissemination, all of which can constrain early antimicrobial impact and complicate interpretation of short-interval CFU readouts (20, 42, 43). Consistent with these distinctions, exposure-dependent reductions in pulmonary neutrophil recruitment are observed here in EMRSA-15 pneumonia but not in *Klebsiella* infection despite identical peptide dosing. Importantly, although simple, pathogen-independent, direct immunomodulation appears unlikely, this divergence does not fully exclude immunomodulation as it could be consistent with host-defense peptides exerting context-dependent effects—via pathogen-associated molecular patterns interactions (e.g., lipopolysaccharide/lipoteichoic acid), modulation of inflammatory signaling pathways, and/or altered leukocyte trafficking—whose detectability depends on infection stage and when the readout is taken (3, 4, 20). Finally, the inoculum effect provides a direct bridge between the *in vitro* concentration requirement and *in vivo* context. AMP activity can be largely stable across moderate inocula but shift at higher densities, making early efficacy more sensitive to burden, compartmentalization, and microenvironment (44, 45). Together, these findings support the view that early immune and microbiological readouts in antimicrobial-peptide therapy must be interpreted within an integrated pharmacokinetic–pathogen–host framework, consistent with PK/PD modeling perspectives that emphasize exposure and context as determinants of *in vivo* outcome (46).

Interpretation of the whole-body imaging data should nevertheless recognize that the IRDye 800CW conjugates used for biodistribution studies possess altered physicochemical properties relative to the parent peptides (Table 3), and the measured fluorescence cannot distinguish intact peptide from labeled metabolites. The imaging experiments therefore provide comparative information on the relative disposition of identically labeled l- and d-enantiomers rather than absolute pharmacokinetic measurements of the unmodified therapeutic peptide. Nonetheless, taken together, these data indicate that the principal barrier to rapid *in vivo* efficacy of systemically delivered pleurocidin-derived peptides is insufficient and short-lived exposure at sites of infection. Multiple studies show that even antimicrobial peptides with demonstrable *in vivo* activity—illustrated by rhesus theta-defensin-1, the antibacterial peptide NZX, and the plectasin-derived peptide NZ2114—have pharmacokinetics characterized by rapid clearance and/or efficacy that depends on achieving and maintaining adequate exposure, often through dosing strategies that sustain exposure over time (47–50). These constraints provide a mechanistic framework for interpreting the dissociation observed here between rapid *in vitro* killing, early modulation of pulmonary leukocyte recruitment, and the absence of detectable reductions in bacterial burden at early time points.

There is precedent for addressing exposure limitation via exposure-modifying strategies used more broadly across therapeutic peptides. These include stability engineering, reduction of renal clearance by increasing effective molecular size or plasma protein binding, and encapsulation in delivery vehicles—yet for antibacterial AMPs, these approaches remain comparatively underexplored and frequently introduce trade-offs that can reduce antimicrobial activity (51, 52). Consistent with this, published examples in the AMP field include lipid conjugation and nanomedicine approaches that can alter distribution and, in selected settings, improve therapeutic performance (53, 54). In parallel, biodistribution studies of radiolabeled host-defense peptides (including LL-37) and radiolabeled lactoferrin-derived peptides demonstrate that tissue distribution and clearance pathways can vary markedly between peptides and formulations, reinforcing the need for exposure-aware optimization rather than reliance on intrinsic potency alone (15, 16, 19).

Finally, the divergence observed here between early inflammatory modulation and microbiological endpoints underscores the importance of time-resolved pharmacodynamic assessment, because treatment-linked changes in inflammatory proteomes/cytokine trajectories and leukocyte recruitment can precede, or occur independently of, measurable reductions in bacterial burden (25, 36). Collectively, these findings support a translational strategy in which pleurocidin-derived peptides are advanced through PK-informed systemic dosing, improved control of whole-body exposure through formulation where needed, and appropriately timed efficacy/immune readouts, rather than being judged solely on early CFU reduction following bolus administration.

### Conclusion

In conclusion, this study demonstrates that the early *in vivo* behavior of systemically administered pleurocidin-derived antimicrobial peptides is governed predominantly by exposure kinetics, stereochemistry, and infection context rather than intrinsic bactericidal potency alone. Although d-P-KR exhibits rapid, concentration-dependent killing across multiple pathogens *in vitro*, substantially outperforming even aminoglycosides in this regard, enantiomer-dependent biodistribution and rapid clearance constrain effective exposure at sites of infection following systemic dosing, such that this performance does not yet translate *in vivo*. Modifying delivery strategy through higher dose infusion prolongs systemic exposure and is sufficient to elicit early, exposure-dependent reductions in pulmonary neutrophil recruitment despite the absence of detectable early CFU reduction. Consequently, the divergence between inflammatory and microbiological endpoints, and between localized and disseminating infection models, reinforces the importance of time-resolved and context-aware efficacy readouts. Collectively, these findings support a translational framework in which PK-informed dosing, stereochemical optimization, and exposure-focused formulation are prioritized alongside antibacterial potency when advancing antimicrobial peptides for systemic therapeutic use.

## SUPPLEMENTAL MATERIAL

Supplemental Material mbio.01718-26-s0001.pdf Supplemental schemes, tables, and figures.

ASM does not own the copyrights to Supplemental Material that may be linked to, or accessed through, an article. The authors have granted ASM a non-exclusive, world-wide license to publish the Supplemental Material files. Please contact the corresponding author directly for reuse.
