# Supplementary material for: Enantiomer-dependent biodistribution and exposure determine the in vivo behavior of a pleurocidin-derived antimicrobial peptide
Source: mBio. 2026 Aug 28;17(10):e01718-26. doi: 10.1128/mbio.01718-26 (PMC13712954; doi:10.1128/mbio.01718-26)
Supplement: Supplementary file 1 [file mbio.01718-26-s0001.pdf]

Enantiomer-dependent biodistribution and exposure determine the *in vivo* behaviour of a pleurocidin-derived antimicrobial peptide

Janis Romanopulos[1], Paul Cressey[1], Charlotte K. Hind[2], Seong-Heun Kim[1], Zayed Shalim[1], Nadiya Mohamed Akram,[1] Hala Alhadi[1], Amelia Claxton[1], Yuang You[1], Anna V. Kelis[1], Bailey Ramsden[1], Siham Memdouh[1], Tam T. Bui[3], Simon J. Cleary[1], Maya Thanou[1], Richard T. Amison[1], Vincenzo Abbate[1] J. Mark Sutton[2] and A. James Mason[1]*

[1]Institute of Pharmaceutical Science, School of Cancer & Pharmaceutical Sciences, King's College London, Franklin-Wilkins Building, 150 Stamford Street, London, SE1 9NH, United Kingdom

[2]Antimicrobial Discovery, Development and Diagnostics (AD[3]), Countermeasures Development, Evaluation and Preparedness, UK Health Security Agency, Porton Down Salisbury, UK

[3]Centre for Biomolecular Spectroscopy and Randall Division of Cell and Molecular Biophysics, King's College London, London SE1 1UL

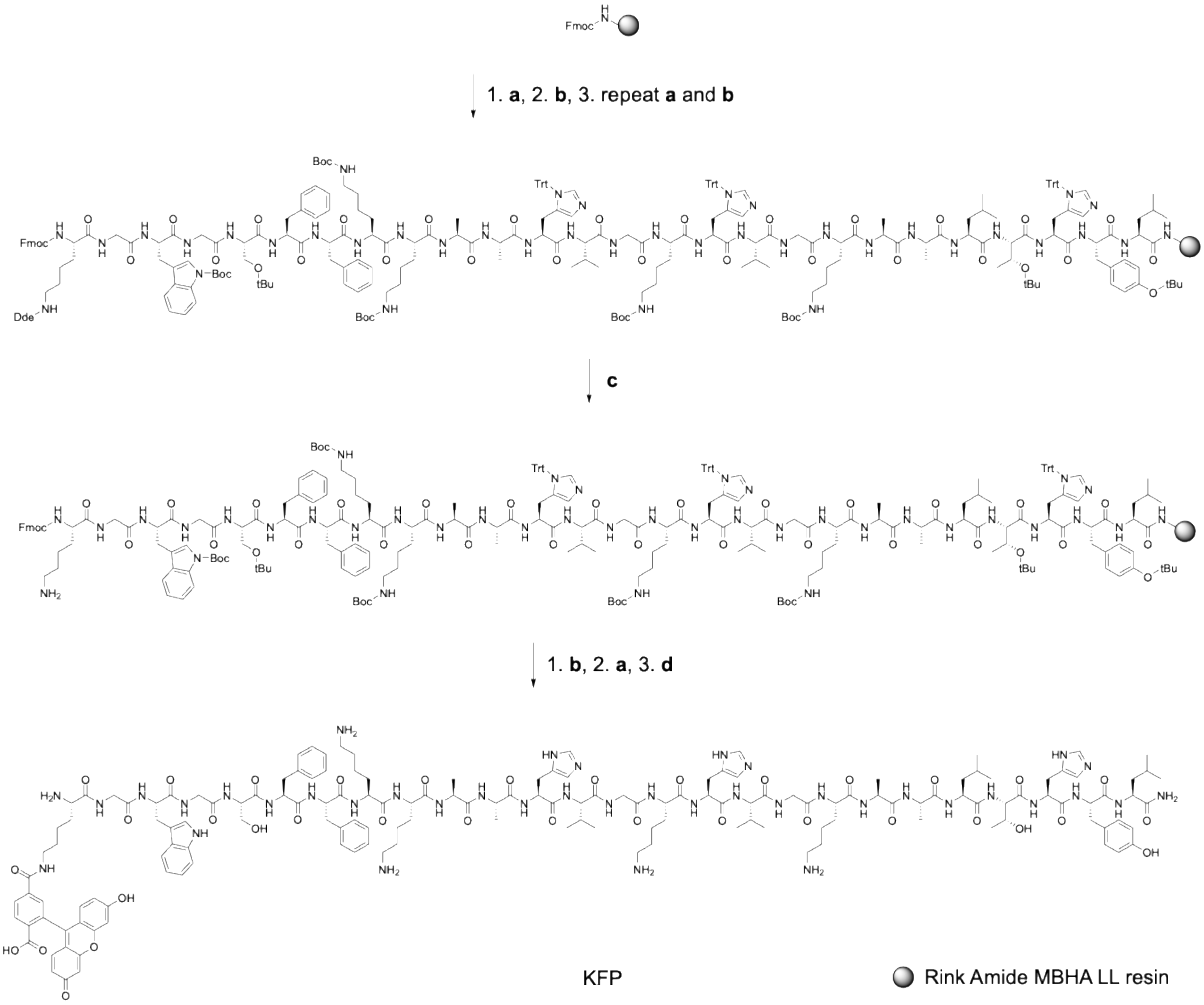

Supplementary Scheme S1. Synthesis of KFP, exemplifying the synthetic strategy employed for the 6-FAM-labelled analogues of pleurocidin, pleurocidin-KR and their enantiomers. a – 20% (v/v) piperidine in DMF, 4 × 15 min; **b** – 0.25 M Fmoc-aa-OH or 6-FAM (4 equiv), 0.5 M Oxyma Pure (4 equiv) and 0.5 M DIC (4 equiv) in NMP, 10 min, 75˚C (microwave heating); **c** – 15 mL/g 0.3 µM hydroxylamine hydrochloride, 0.225 µM imidazole in 5:1 (v/v) NMP/DCM, 3 × 3 h; **d** – 15 mL/g 95:2.5:2.5 (v/v/v) TFA/TIPS/water, 3 h.

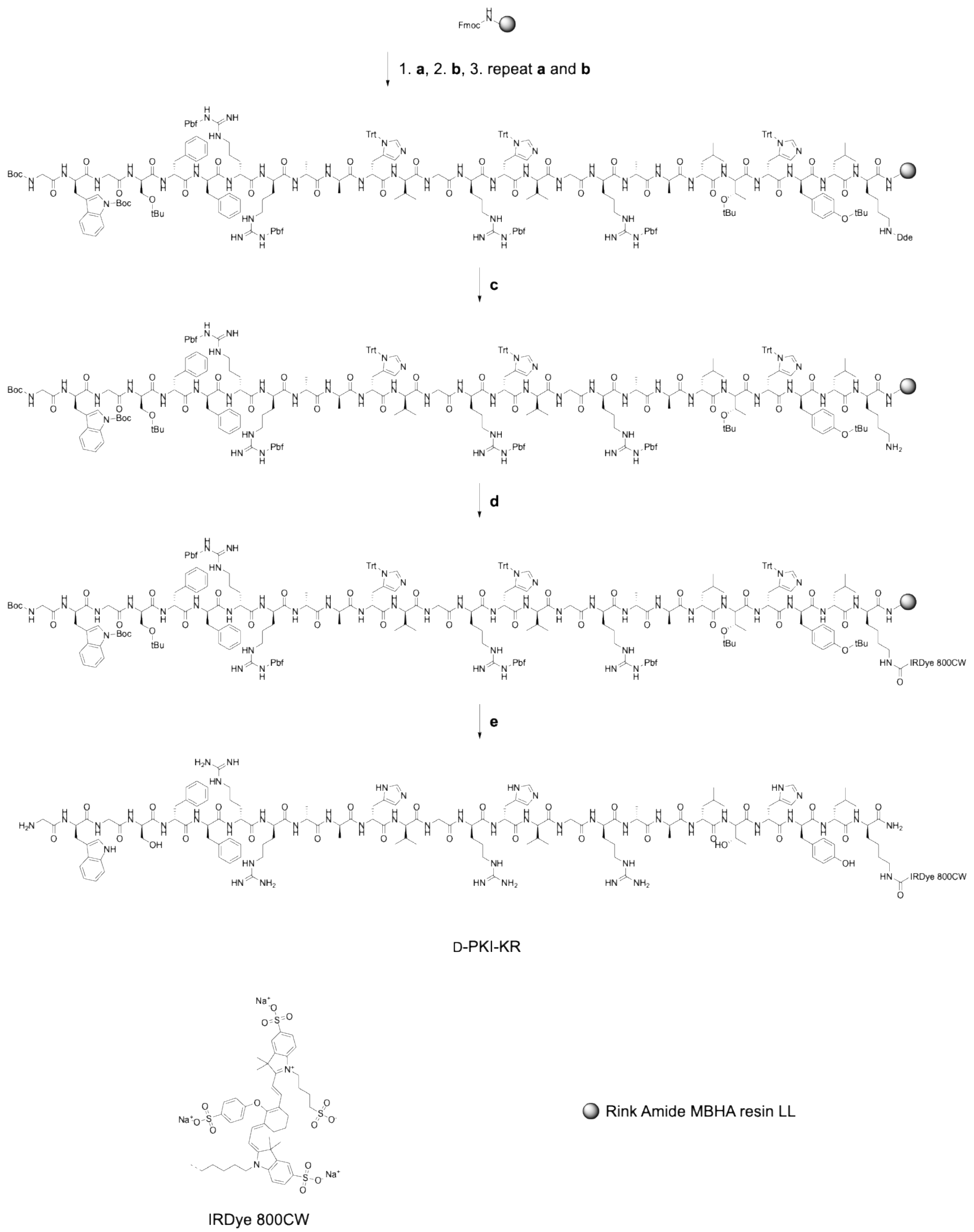

Supplementary Scheme S2. Synthesis of D-PKI-KR, exemplifying the synthetic strategy employed for the IRDye 800CW-labelled analogue of pleurocidin-KR and its enantiomer. **a** – 20% (v/v) piperidine in DMF, 4 × 15 min; **b** – 0.25 M Fmoc-aa-OH or Boc-aa-OH (4 equiv), 0.5 M Oxyma Pure (4 equiv) and 0.5 M DIC (4 equiv) in NMP, 10 min, 75˚C (microwave heating); **c** – 15 mL/g 0.3 μM hydroxylamine hydrochloride, 0.225 μM imidazole in 5:1 (v/v) NMP/DCM, 3 × 3 h; **d** – 0.05 M IRDye 800CW NHS ester (1.2 equiv), DIPEA (1.5 equiv) in DMF, anhydrous, 6 h, **e** – 15 mL/g 90:5:3:2 (v/v/w/v) TFA/thioanisole/DTT/anisole, 2 h.

Supplementary Table S1. Minimum inhibitory concentration (MIC) of pleurocidin, its enantiomer and their 6-FAM-labelled analogues against selected Gram-negative and Gram-positive bacteria in Mueller Hinton broth (MHB). The fold change compared to the corresponding value of the parent peptide shown in round brackets and colour-coded – <4-fold change – green, 4-fold change – orange and >4-fold change – red.

Panel	Strain	MIC in MHB [µg/mL] (fold change compared to the parent)					
		P	KFP	PKF	D-P	D-KFP	D-PKF
Gram-negative	*A. baumannii* AYE	2	4 (2×)	4 (2×)	2	64 (32×)	1 (0.5×)
	A. baumannii ATCC 17978	1	2 (2×)	2 (2×)	1	64 (64×)	1 (1×)
	E. coli NCTC 12923	2	2 (1×)	4 (2×)	1	32 (32×)	2 (2×)
	K. pneumoniae M6	4	2 (0.5×)	8 (2×)	2	32 (16×)	4 (2×)
	K. pneumoniae NCTC 13368	4	16 (4×)	8 (2×)	2	32 (16×)	4 (2×)
	P. aeruginosa PAO1	4	16 (4×)	8 (2×)	2	32 (16×)	4 (2×)
	P. aeruginosa NCTC 13437	16	64 (4×)	16 (1×)	8	64 (8×)	8 (1×)
Gram-positive	*En. faecalis* NCTC 775	64	64 (1×)	128 (2×)	32	128 (4×)	128 (4×)
	En. faecalis NCTC 12201	128	64 (0.5×)	128 (1×)	128	128 (1×)	128 (1×)
	En. faecium NCTC 12204	128	64 (0.5×)	128 (1×)	64	128 (2×)	128 (2×)
	S. aureus SA-1199B	32	128 (4×)	32 (1×)	16	64 (4×)	64 (4×)
	S. aureus USA300	16	>128 (>8×)	64 (4×)	16	128 (8×)	64 (4×)
	S. aureus NCTC 6571	8	64 (8×)	8 (1×)	16	32 (2×)	32 (2×)
	S. aureus NCTC 13616	16	>128 (>8×)	64 (4×)	16	128 (8×)	64 (4×)
Extended *P. aeruginosa*	*P. aeruginosa* CAS2	16	32 (2×)	16 (1×)	8	64 (8×)	4 (0.5×)
	P. aeruginosa CAS3	16	64 (4×)	32 (2×)	4	64 (16×)	4 (1×)
	P aeruginosa CAS4	>128	>128 (-)	>128 (-)	8	128 (16×)	8 (1×)
	P. aeruginosa GH12	32	32 (1×)	32 (1×)	4	64 (16×)	4 (1×)
	P. aeruginosa RP73	16	64 (4×)	32 (2×)	16	128 (8×)	16 (1×)
	P. aeruginosa 372261	16	64 (4×)	32 (2×)	4	64 (8×)	4 (1×)

Supplementary Table S2. Minimum inhibitory concentration (MIC) of pleurocidin (P), its enantiomer and their 6-FAM-labelled analogues against selected Gram-negative and Gram-positive bacteria in Roswell Park Memorial Institute 1640 medium supplemented with 5% Mueller Hinton broth (RPMI + MHB). The fold change compared to the corresponding value of the parent peptide shown in round brackets and colour-coded – <4-fold change – green, 4-fold change – orange and >4-fold change – red.

Panel	Strain	MIC in RPMI + MHB [μg/mL] (fold change compared to the parent)					
		P	KFP	PKF	D-P	D-KFP	D-PKF
Gram-negative	*A. baumannii* AYE	8	8 (1×)	8 (1×)	2	32 (16×)	4 (2×)
	A. baumannii ATCC 17978	8	8 (1×)	8 (1×)	2	32 (16×)	4 (2×)
	E. coli NCTC 12923	8	8 (1×)	8 (1×)	2	32 (16×)	4 (2×)
	K. pneumoniae M6	16	16 (1×)	16 (1×)	4	64 (16×)	8 (2×)
	K. pneumoniae NCTC 13368	16	16 (1×)	16 (1×)	4	64 (16×)	16 (4×)
	P. aeruginosa PAO1	>128	>128 (-)	>128 (-)	8	128 (16×)	64 (8×)
	P. aeruginosa NCTC 13437	>128	>128 (-)	128 (-)	16	128 (8×)	32 (2×)
Gram-positive	*En. faecalis* NCTC 775	32	64 (2×)	64 (2×)	4	128 (32×)	32 (8×)
	En. faecalis NCTC 12201	64	128 (2×)	128 (2×)	64	128 (2×)	64 (1×)
	En. faecium NCTC 12204	>128	>128 (-)	128 (-)	64	128 (2×)	128 (2×)
	S. aureus SA-1199B	8	128 (16x)	16 (2×)	4	64 (16×)	64 (16×)
	S. aureus USA300	16	>128 (>8×)	32 (2×)	8	128 (16×)	16 (2×)
	S. aureus NCTC 6571	16	128 (8×)	8 (0.5x)	4	32 (8×)	8 (2×)
	S. aureus NCTC 13616	16	128 (8×)	32 (2×)	4	64 (16×)	16 (4×)
Extended *P. aeruginosa*	*P. aeruginosa* CAS2	>128	>128 (-)	>128 (-)	16	128 (8×)	64 (4×)
	P. aeruginosa CAS3	>128	>128 (-)	>128 (-)	16	128 (8×)	32 (2×)
	P aeruginosa CAS4	>128	>128 (-)	>128 (-)	32	>128 (>4x)	64 (2×)
	P. aeruginosa GH12	>128	>128 (-)	>128 (-)	16	128 (8×)	32 (2×)
	P. aeruginosa RP73	128	128 (1×)	128 (1×)	16	>128 (>8×)	64 (4×)
	P. aeruginosa 372261	>128	>128 (-)	>128 (-)	8	128 (16×)	32 (4×)

Supplementary Table S3. Antimicrobial activity of D-pleurocidin-KR (D-P-KR) and its 6-FAM-labelled analogues in Mueller Hinton broth (MHB) and Roswell Park Memorial Institute 1640 medium supplemented with 5% Mueller Hinton broth (RPMI + MHB). The fold change compared to the corresponding value of the parent peptide shown in round brackets and colour-coded – <4-fold change – green, 4-fold change – orange and >4-fold change – red.

Panel	Strain	MIC [µg/mL] (fold change compared to the parent)			
		MHB		RPMI + MHB	
		D-P-KR	D-PKF-KR	D-P-KR	D-PKF-KR
Gram-negative	*A. baumannii* AYE	2	2 (1×)	2	4 (2×)
	A. baumannii ATCC 17978	2	4 (2×)	2	4 (2×)
	E. coli NCTC 12923	1	2 (2×)	2	4 (2×)
	K. pneumoniae M6	4	4 (1×)	2	8 (4×)
	K. pneumoniae NCTC 13368	2	4 (2×)	2	16 (8×)
	P. aeruginosa PAO1	4	4 (1×)	4	32 (8×)
	P. aeruginosa NCTC 13437	4	16 (4×)	8	32 (4×)
Gram-positive	*En. faecalis* NCTC 775	8	32 (4×)	2	16 (8x)
	En. faecalis NCTC 12201	16	128 (8×)	16	32 (2×)
	En. faecium NCTC 12204	16	128 (8×)	16	32 (2×)
	S. aureus SA-1199B	8	64 (8×)	4	4 (1×)
	S. aureus USA300	8	64 (8×)	4	8 (2×)
	S. aureus NCTC 6571	8	32 (4×)	2	4 (2×)
	S. aureus NCTC 13616	8	64 (8×)	2	8 (4×)
Extended *P. aeruginosa*	*P. aeruginosa* CAS2	2	8 (4×)	8	32 (4×)
	P. aeruginosa CAS3	2	16 (4×)	8	16 (2×)
	P aeruginosa CAS4	8	16 (2×)	16	64 (4×)
	P. aeruginosa GH12	2	8 (4×)	8	32 (4×)
	P. aeruginosa RP73	8	32 (4×)	16	32 (2×)
	P. aeruginosa 372261	2	8 (4×)	8	32 (4×)

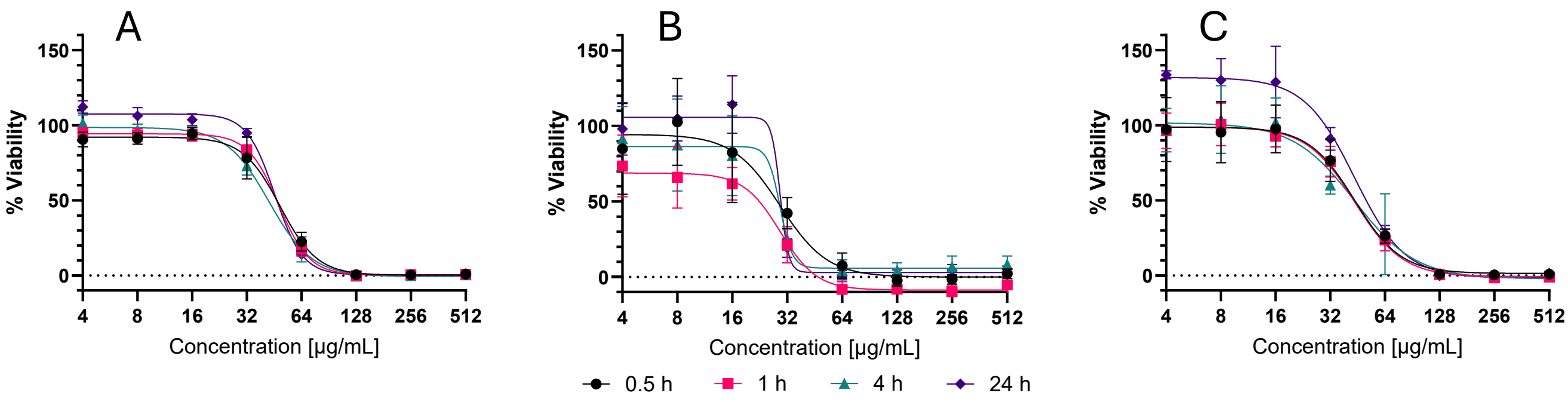

Supplementary Figure S1. Time resolved toxicity of D-P-KR to human cells. MTT assay dose-response curves showing the effect of 0.5, 1, 4 and 24 h D-P-KR exposure on the viability of lung A549 (A), kidney HEK293T (B) and liver Hep G2 (C) cells. Data presented as means (n = 6) ± standard deviation (SD).

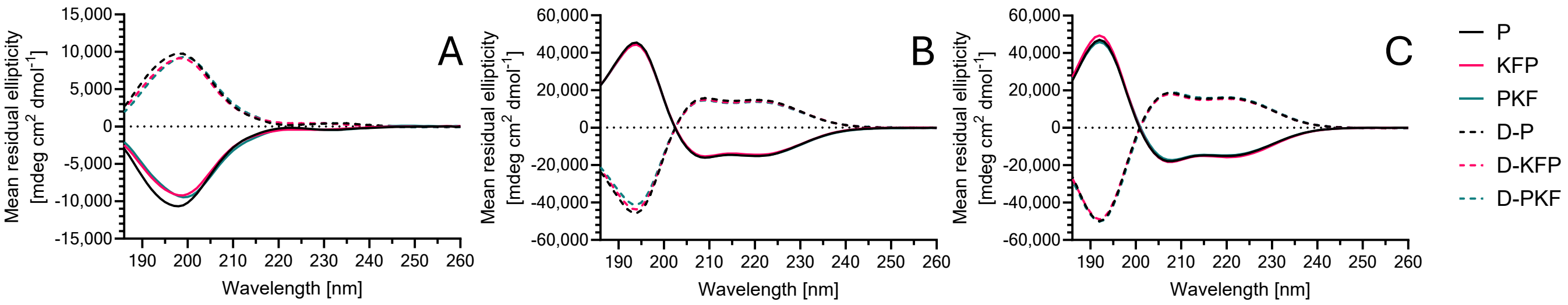

Supplementary Figure S2. Impact of 6-FAM labelling on pleurocidin and D-pleurocidin peptide conformations. Circular dichroism spectra of pleurocidin (P), its enantiomer and their 6-FAM-labelled analogues in 5 mM Tris buffer pH 7.0 (A), 5 mM Tris buffer with SDS (B) and TFE (C) at 23˚C.

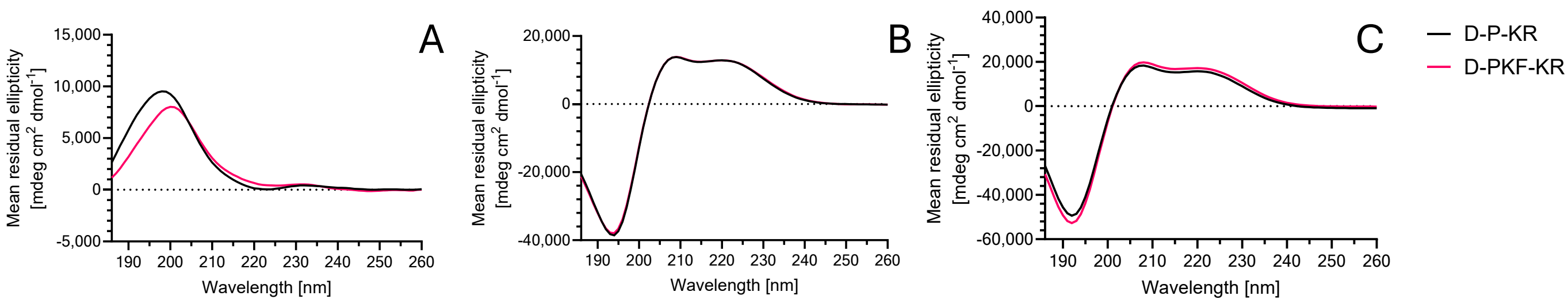

Supplementary Figure S3. Impact of 6-FAM labelling on D-P-KR conformations. Circular dichroism spectra of D-pleurocidin-KR (D-P-KR) and its 6-FAM-labelled analogue D-PKF-KR in 5 mM Tris buffer pH 7.0 (A), 5 mM Tris buffer with SDS (B) and TFE (C) at 23˚C.

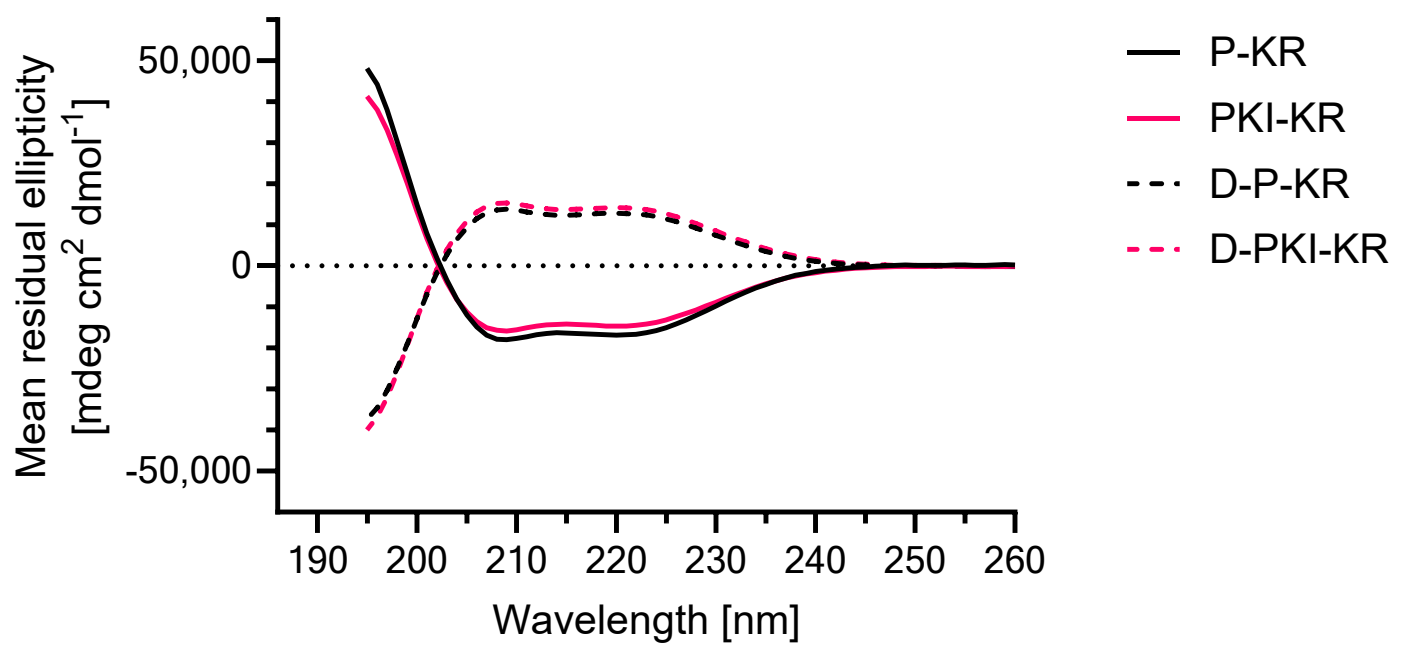

Supplementary Figure S4. Impact of IRDye-800CW labelling on D-P-KR conformation. Circular dichroism spectra of pleurocidin-KR (P-KR), D-pleurocidin (D-P-KR) and their IRDye 800CW-labelled analogues PKI-KR and D-PKI-KR in 5 mM Tris buffer pH 7.0 with SDS at 23˚C.

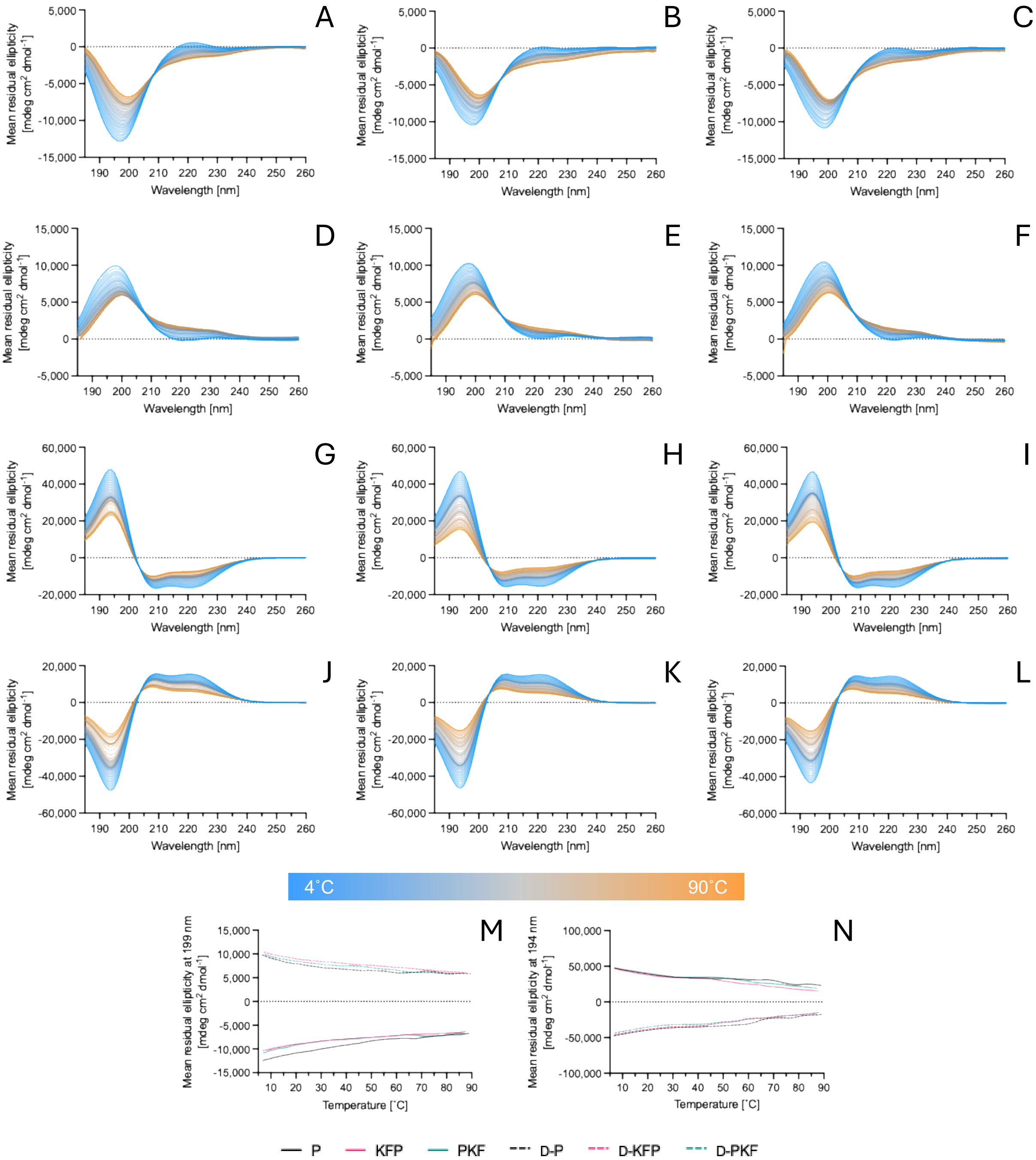

Supplementary Figure S5. Impact of 6-FAM labelling on pleurocidin and D-pleurocidin temperature dependent peptide conformations. Circular dichroism spectra of pleurocidin (P; A and G), D-pleurocidin (D-P; D and J) and their 6-FAM-labelled analogues KFP (B and H), D-KFP (E and K), PKF (C and I) and D-PKF (F and L) taken between 4 and 90˚C in 5 mM Tris buffer pH 7.0 (A-F) and Tris buffer with SDS (G-L) with mean residual ellipticity values at 199 nm (Tris buffer conditions, M) and at 194 nm (Tris buffer with SDS conditions, N).

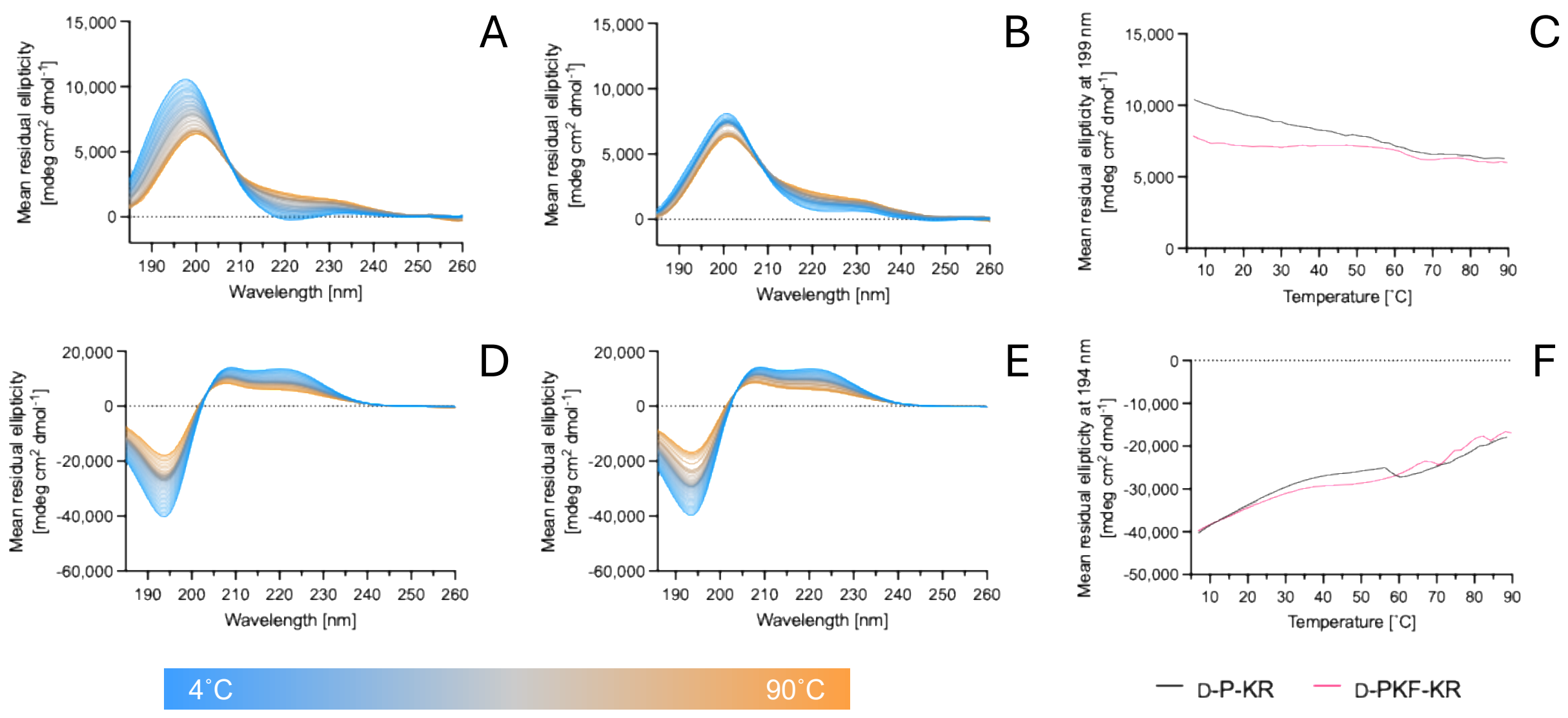

Supplementary Figure S6. Impact of 6-FAM labelling on D-P-KR temperature dependent conformations. Circular dichroism spectra of D-pleurocidin-KR (D-P-KR; A and D) and its 6-FAM-labelled analogue D-PKF-KR (B and E) taken between 4 and 90˚C in 5 mM Tris buffer pH 7.0 (A and B) and Tris buffer with SDS (D and E) with mean residual ellipticity values at 199 nm (Tris buffer conditions, C) and at 194 nm (Tris buffer with SDS conditions, D).

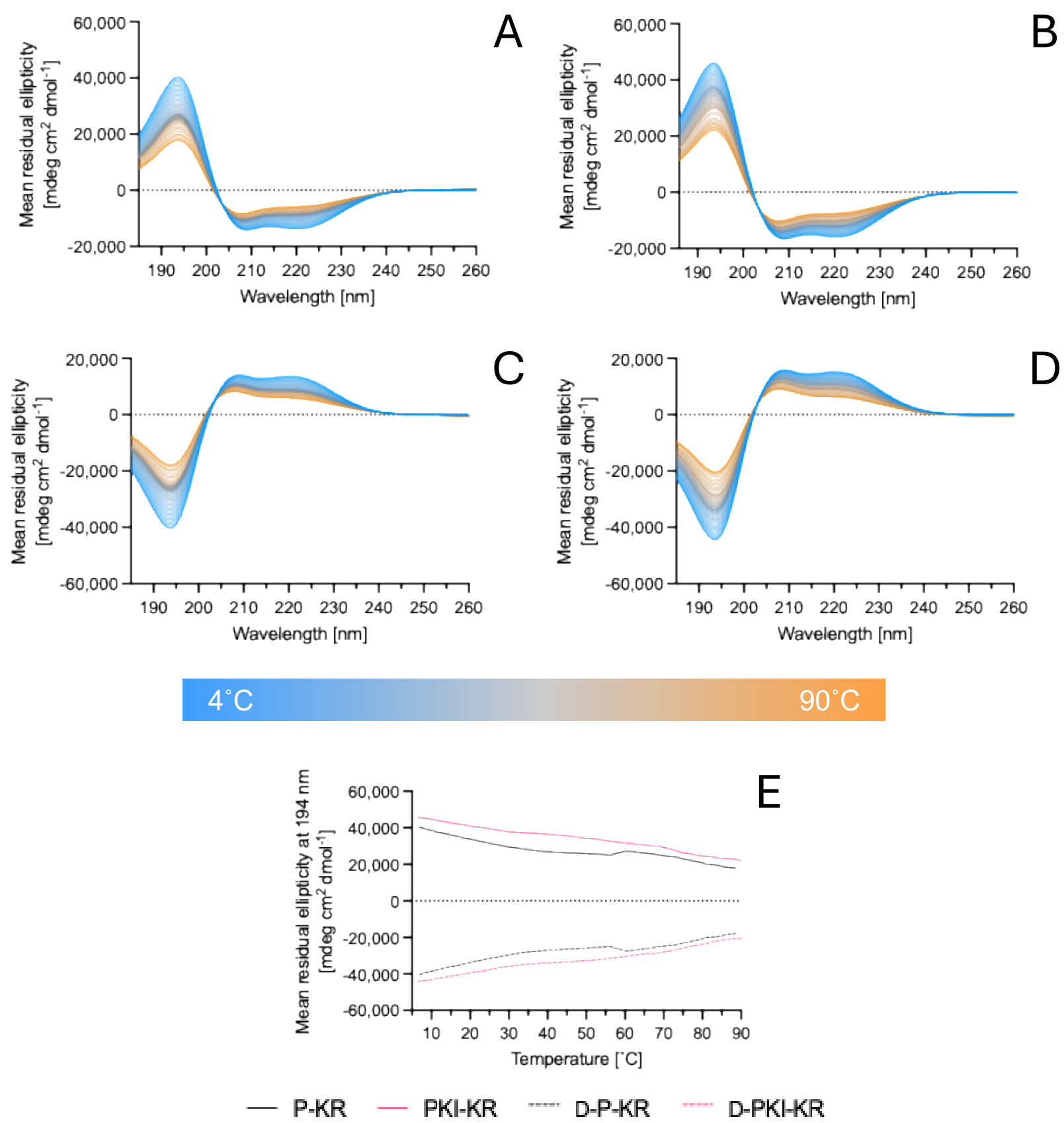

Supplementary Figure S7. Impact of IRDye-800CW labelling on D-PKR temperature dependent conformations. Circular dichroism spectra of pleurocidin-KR (P-KR; A), D-pleurocidin-KR (D-P-KR; D) and their IRDye-800CW labelled analogues PKI-KR (B) and D-PKI-KR (D) taken between 4 and 90˚C in Tris buffer with SDS with mean residual ellipticity values at 194 nm (E). The spectra of P-KR were obtained by inverting those of D-P-KR along the x-axis.

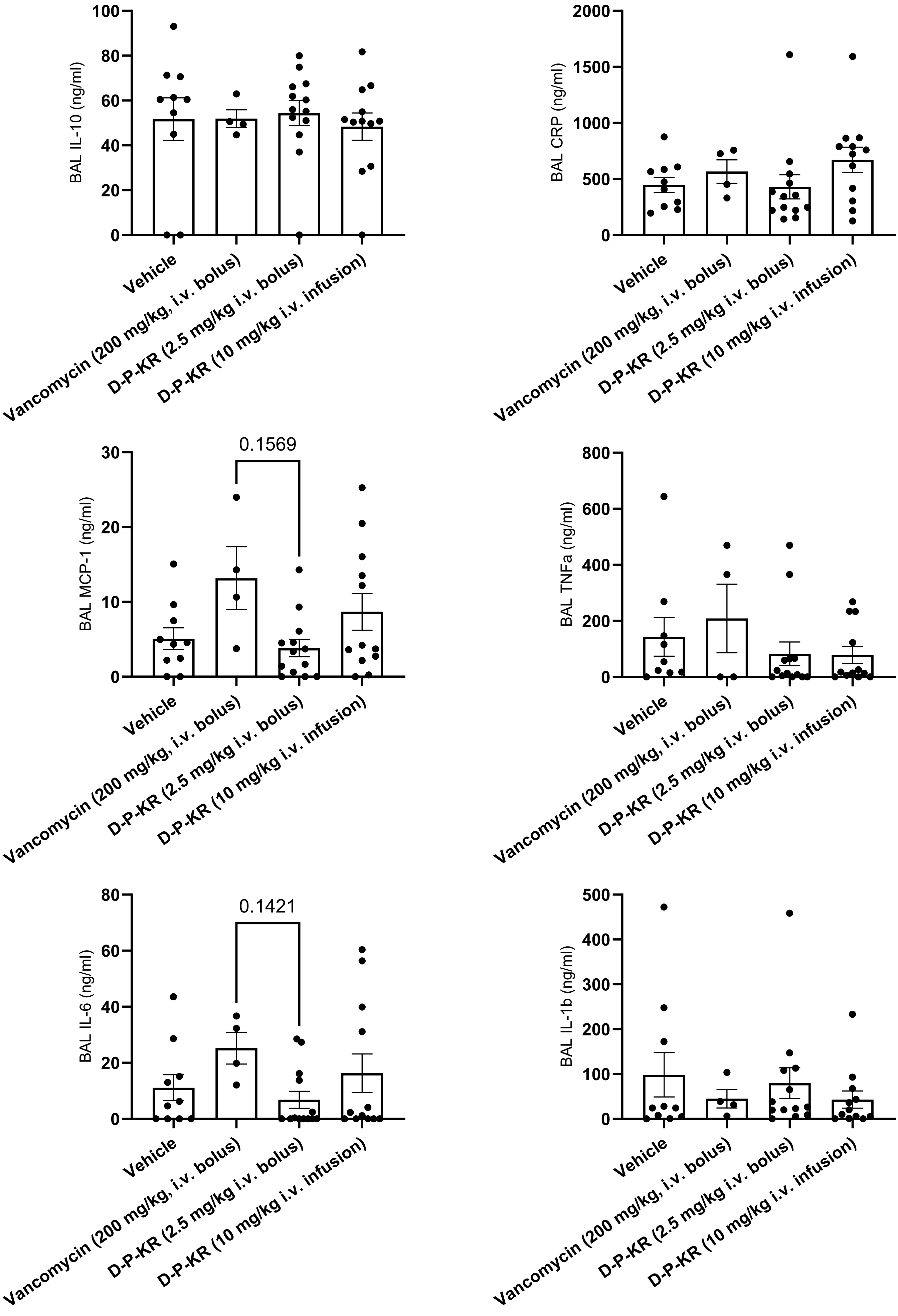

Supplementary Figure S8. Cytokine response in bronchoalveolar lavage (BAL) fluid for *Staphylococcus aureus* pneumonia with indicated therapy. Enzyme-linked immunosorbent assay (ELISA) data according to therapy of C57Bl/6 mice at 3 h p.i. infected with EMRSA-15. Comparisons are Kruskal-Wallis test according to data normality with only comparisons $p \leq 0.2$ shown. Data is shown as mean ± SEM.

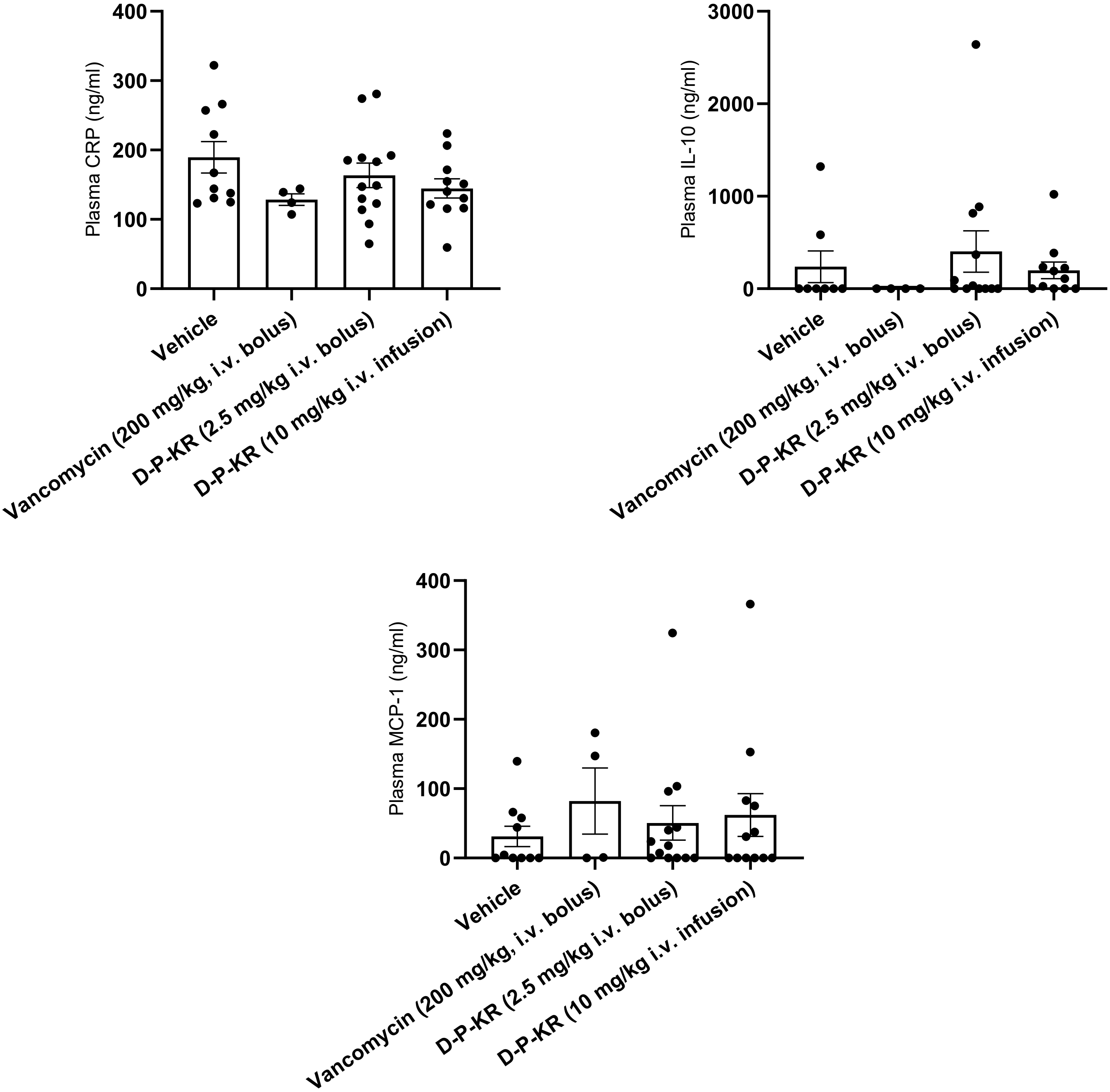

Supplementary Figure S9. Cytokine response in plasma for *Staphylococcus aureus* pneumonia with indicated therapy. Enzyme-linked immunosorbent assay (ELISA) data according to therapy of C57Bl/6 mice at 3 h p.i. infected with EMRSA-15. Data is shown as mean ± SEM.

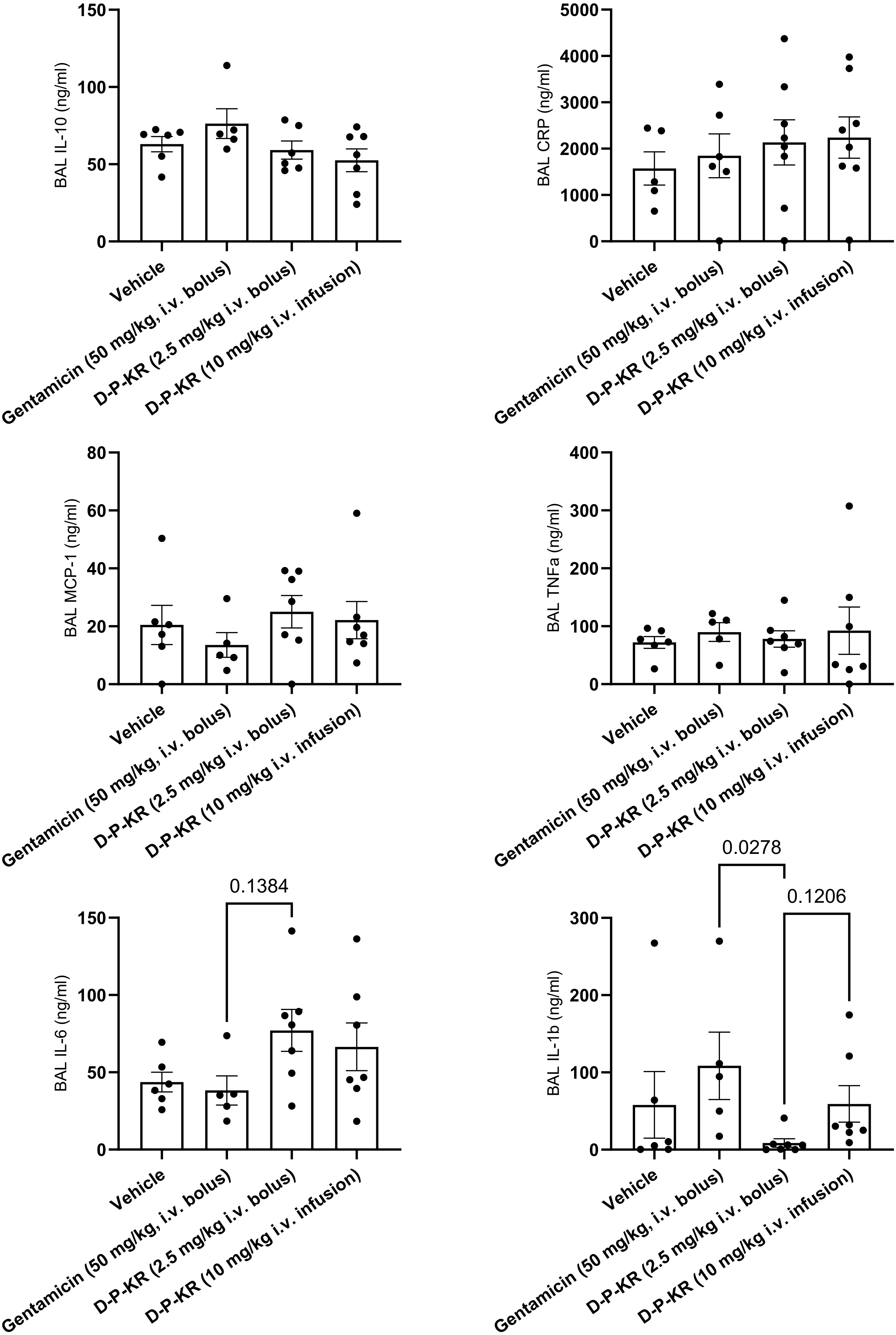

Supplementary Figure S10. Cytokine response in bronchoalveolar lavage (BAL) fluid for *Klebsiella pneumoniae* pneumonia with indicated therapy. Enzyme-linked immunosorbent assay (ELISA) data according to therapy of C57Bl/6 mice at 26 h p.i. infected with delafloxacin resistant *K. pneumoniae*. Comparisons are Kruskal-Wallis test or Lognormal ordinary one-way ANOVA according to data normality with only comparisons $p \leq 0.2$ shown. Data is shown as mean ± SEM.

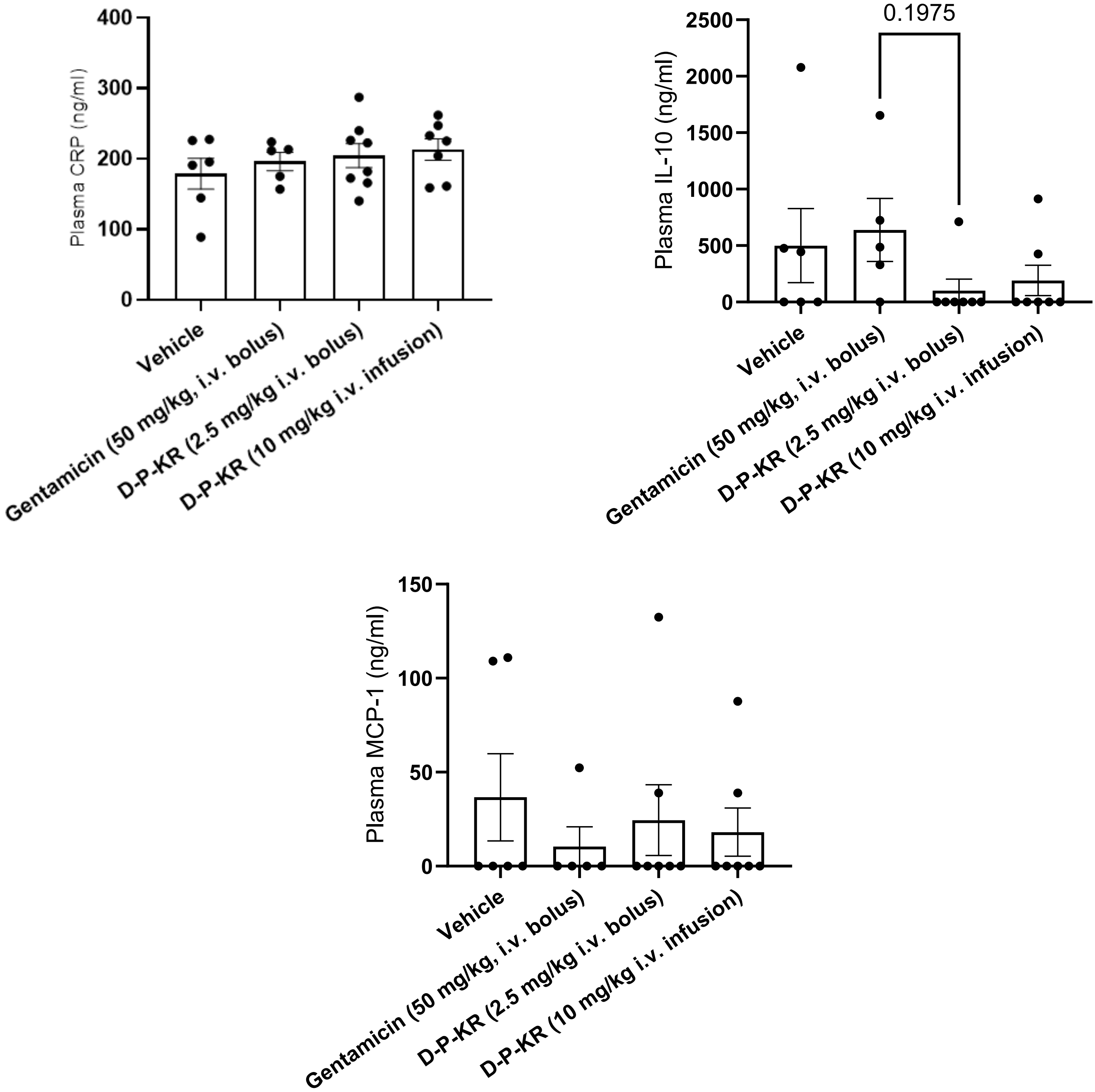

Supplementary Figure S11. Cytokine response in plasma for *Klebsiella pneumoniae* pneumonia with indicated therapy. Enzyme-linked immunosorbent assay (ELISA) data according to therapy of C57Bl/6 mice at 26 h p.i. infected with delafloxacin resistant *K. pneumoniae*. Comparisons are Kruskal-Wallis test according to data normality with only comparisons $p \leq 0.2$ shown. Data is shown as mean ± SEM.
